# A faux hawk fullerene with PCBM-like properties†
†Electronic supplementary information (ESI) available: Additional figures and tables described in the text. CSD-428507. For ESI and crystallographic data in CIF or other electronic format see DOI: 10.1039/c4sc02970d



**DOI:** 10.1039/c4sc02970d

**Published:** 2014-12-16

**Authors:** Long K. San, Eric V. Bukovsky, Bryon W. Larson, James B. Whitaker, S. H. M. Deng, Nikos Kopidakis, Garry Rumbles, Alexey A. Popov, Yu-Sheng Chen, Xue-Bin Wang, Olga V. Boltalina, Steven H. Strauss

**Affiliations:** a Department of Chemistry , Colorado State University , Fort Collins , CO 80523 , USA . Email: olga.boltalina@colostate.edu ; Email: steven.strauss@colostate.edu; b National Renewable Energy Laboratory , Golden , CO 80401 , USA . Email: nikos.kopidakis@nrel.gov ; Email: garry.rumbles@nrel.gov; c Physical Sciences Division , Pacific Northwest National Laboratory , MS K8-88, P.O. Box 999 , Richland , WA 99352 , USA . Email: xuebin.wang@pnnl.gov; d Leibniz Institute for Solid State and Materials Research , 01069 Dresden , Germany . Email: a.popov@ifw-dresden.de; e ChemMatCARS Beamline , University of Chicago Advanced Photon Source , Argonne , IL 60439 , USA . Email: yschen@cars.uchicago.edu

## Abstract

Reaction of C_60_, C_6_F_5_CF_2_I, and SnH(*n*-Bu)_3_ produced, among other unidentified fullerene derivatives, the two new compounds 1,9-C_60_(CF_2_C_6_F_5_)H (**1**) and 1,9-C_60_(*cyclo*-CF_2_(2-C_6_F_4_)) (**2**). The highest isolated yield of **1** was 35% based on C_60_. Depending on the reaction conditions, the relative amounts of **1** and **2** generated *in situ* were as high as 85% and 71%, respectively, based on HPLC peak integration and summing over all fullerene species present other than unreacted C_60_. Compound **1** is thermally stable in 1,2-dichlorobenzene (oDCB) at 160 °C but was rapidly converted to **2** upon addition of Sn_2_(*n*-Bu)_6_ at this temperature. In contrast, complete conversion of **1** to **2** occurred within minutes, or hours, at 25 °C in 90/10 (v/v) PhCN/C_6_D_6_ by addition of stoichiometric, or sub-stoichiometric, amounts of proton sponge (PS) or cobaltocene (CoCp_2_). DFT calculations indicate that when **1** is deprotonated, the anion C_60_(CF_2_C_6_F_5_)^–^ can undergo facile intramolecular S_N_Ar annulation to form **2** with concomitant loss of F^–^. To our knowledge this is the first observation of a fullerene-cage carbanion acting as an S_N_Ar nucleophile towards an aromatic C–F bond. The gas-phase electron affinity (EA) of **2** was determined to be 2.805(10) eV by low-temperature PES, higher by 0.12(1) eV than the EA of C_60_ and higher by 0.18(1) eV than the EA of phenyl-C_61_-butyric acid methyl ester (PCBM). In contrast, the relative *E*_1/2_(0/–) values of **2** and C_60_, –0.01(1) and 0.00(1) V, respectively, are virtually the same (on this scale, and under the same conditions, the *E*_1/2_(0/–) of PCBM is –0.09 V). Time-resolved microwave conductivity charge-carrier yield × mobility values for organic photovoltaic active-layer-type blends of **2** and poly-3-hexylthiophene (P3HT) were comparable to those for equimolar blends of PCBM and P3HT. The structure of solvent-free crystals of **2** was determined by single-crystal X-ray diffraction. The number of nearest-neighbor fullerene–fullerene interactions with centroid···centroid (⊙···⊙) distances of ≤10.34 Å is significantly greater, and the average ⊙···⊙ distance is shorter, for **2** (10 nearest neighbors; ave. ⊙···⊙ distance = 10.09 Å) than for solvent-free crystals of PCBM (7 nearest neighbors; ave. ⊙···⊙ distance = 10.17 Å). Finally, the thermal stability of **2** was found to be far greater than that of PCBM.

## Abstract 2

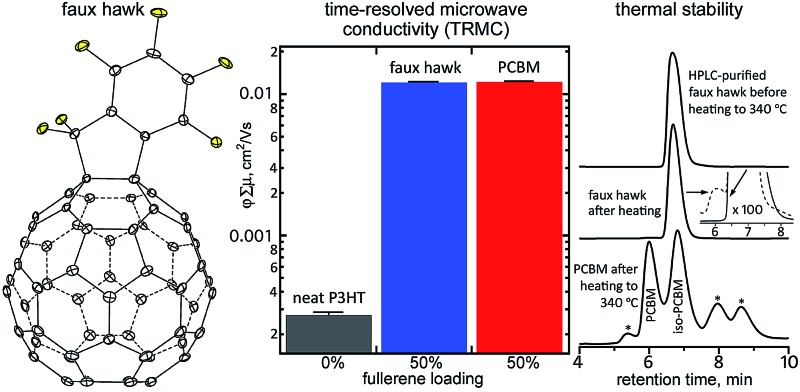
A fluorinated faux hawk fullerene with comparable OPV-relevant TRMC performance and far greater thermal stability than PCBM is reported.

## 1. Introduction

We^1^ and others^2^ have been investigating homoleptic perfluoroalkylfullerenes (PFAFs, fullerene(R_F_)_*n*_) such as 1,7-C_60_(R_F_)_2_ (R_F_ = CF_3_, C_2_F_5_, *n*-C_3_F_7_, *i*-C_3_F_7_, *n*-C_4_F_9_, 2-C_4_F_9_, and *n*-C_8_F_17_),^3,4^ C_74_(CF_3_)_12_,^5^ C_84_(CF_3_)_12_,^2,6,7^ 7,24-C_70_(C_2_F_5_)_2_,^8^ and *C*_3_–C_60_(*i*-C_3_F_7_)_6_ (ref. 9) since 2003. This very large class of fullerene(X)_*n*_ derivatives has fostered an understanding of the relationships between fullerene addition patterns, LUMO shapes and relative energies, perfluoroalkyl chain lengths, and electrochemical/electron affinity properties^4,10,11^ and has afforded a range of structurally similar PFAFs with *E*_1/2_(0/–) values that vary by as much as 0.5 V to be used for fundamental organic photovoltaic (OPV) active-layer studies.^12^ We have recently turned our attention to (i) fullerenes with perfluoroaryl derivatives (*e.g.*, perfluorobenzyl)^13^ and (ii) hydro-PFAFs with one or more H atom substituents,^14,15^ the latter so that their deprotonation and subsequent treatment with electrophiles E^+^ would result in a variety of fullerene(E)(R_F_)_*n*–1_ derivatives for fundamental and applied studies.

**Fig. 1:**
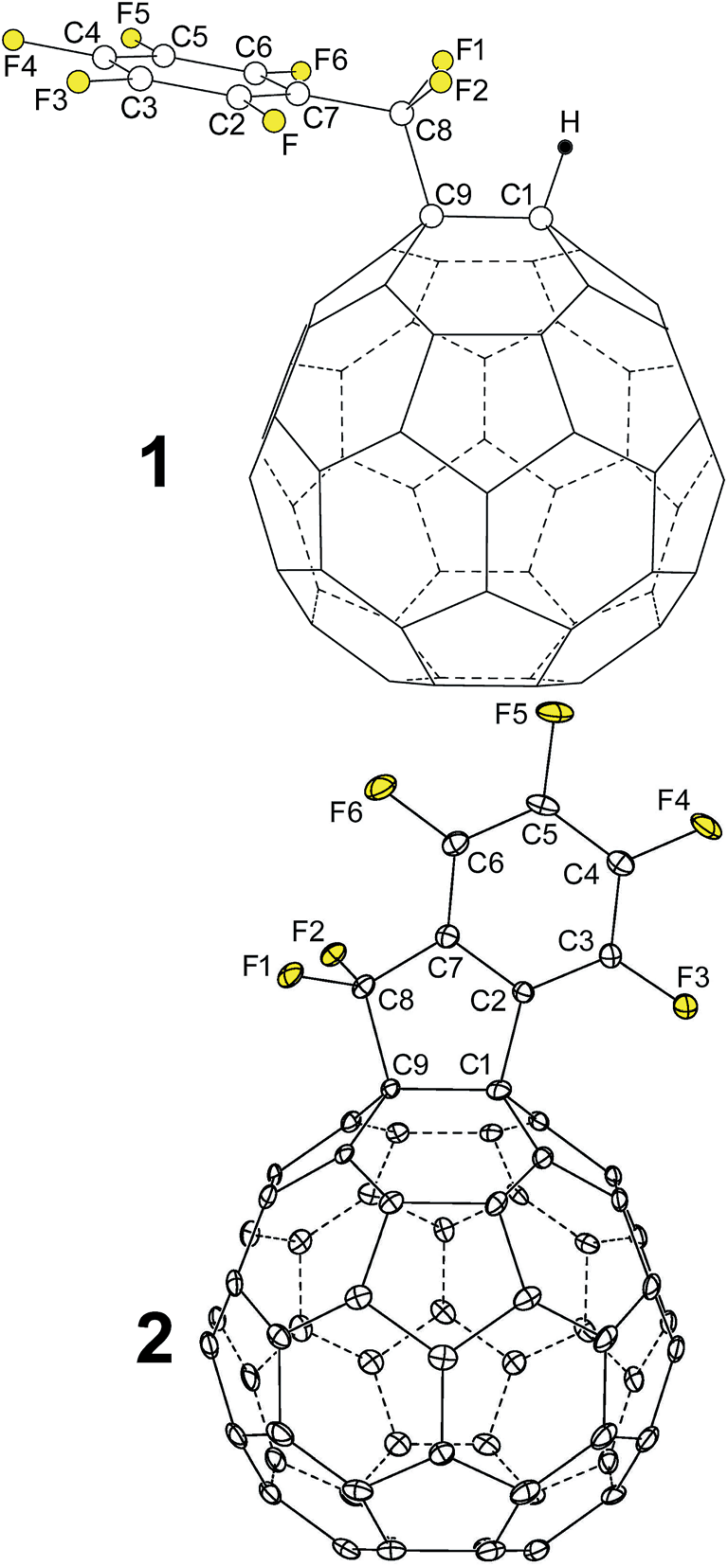
OLYP DFT-optimized structure of 1,9-C_60_(CF_2_C_6_F_5_)H (**1**) and the X-ray structure of 1,9-C_60_(*cyclo*-CF_2_(2-C_6_F_4_)) (**2**; 50% probability ellipsoids). Only the major twin portion of the X-ray structure is shown. The shape of compound **2** is reminiscent of a hairstyle known as the faux hawk, as shown in Fig. S-1.†

We herein report the synthesis of 1,9-C_60_(CF_2_C_6_F_5_)H (**1**), shown in Fig. 1, and its unexpected transformation upon deprotonation or one-electron reduction to the exocyclic “fullerene with a faux hawk” product 1,9-C_60_(*cyclo*-CF_2_(2-C_6_F_4_)) (**2**), also shown in Fig. 1 (see also Fig. S-1; ESI figures and tables, available in the ESI,† are numbered T-1, T-2, S-1, S-2, *etc.*). We propose a reaction sequence for the transformation **1** → **2** + HF that is supported by DFT calculations. The gas-phase electron affinities, solution reduction potentials, thermal stabilities, X-ray diffraction molecular structures, and solid-state packing in solvent-free crystals of **2**, PCBM (phenyl-C_61_-butyric acid methyl ester), and C_60_ are compared and contrasted. Finally, we show that OPV active-layer thin films made from blends of **2** with poly-3-hexylthiophene (P3HT), when studied using time-resolved microwave photoconductivity, exhibit photoinduced charge-carrier yield × mobility figures of merit that rival the OPV active-layer standard blend of PCBM with P3HT, which demonstrates the potential of **2** as an electron acceptor in OPV and other optoelectronic devices.

## 2. Results and discussion

### 2.1. Synthesis of 1,9-C_60_(CF_2_C_6_F_5_)H (**1**) and 1,9-C_60_(*cyclo*-CF_2_(2-C_6_F_4_)) (**2**)

In 1996 Yoshida, Suzuki, and Iyoda reported that the reaction of C_60_, perfluoroalkyliodides (R_F_I), SnH(*n*-Bu)_3_, and a catalytic amount of the radical initiator AIBN in refluxing benzene for 30 h produced 1,9-C_60_(R_F_)H derivatives in moderate yields depending on the ratio of the reagents.^16^ For example, with 12 equiv. *n*-C_6_F_13_I, 5 equiv. SnH(*n*-Bu)_3_, and 0.1 equiv. AIBN (based on C_60_), the yield of 1,9-C_60_(*n*-C_6_F_13_)H was 31% and 64% of the original C_60_ was recovered. With 12 equiv. *n*-C_12_F_25_I, 14 equiv. SnH(*n*-Bu)_3_, and 0.1 equiv. AIBN, the yield of 1,9-C_60_(*n*-C_12_F_25_)H was 26% and 67% of the original C_60_ was recovered. However, no fullerene products containing R_F_ groups were obtained in the absence of AIBN.^16^

**Fig. 2:**
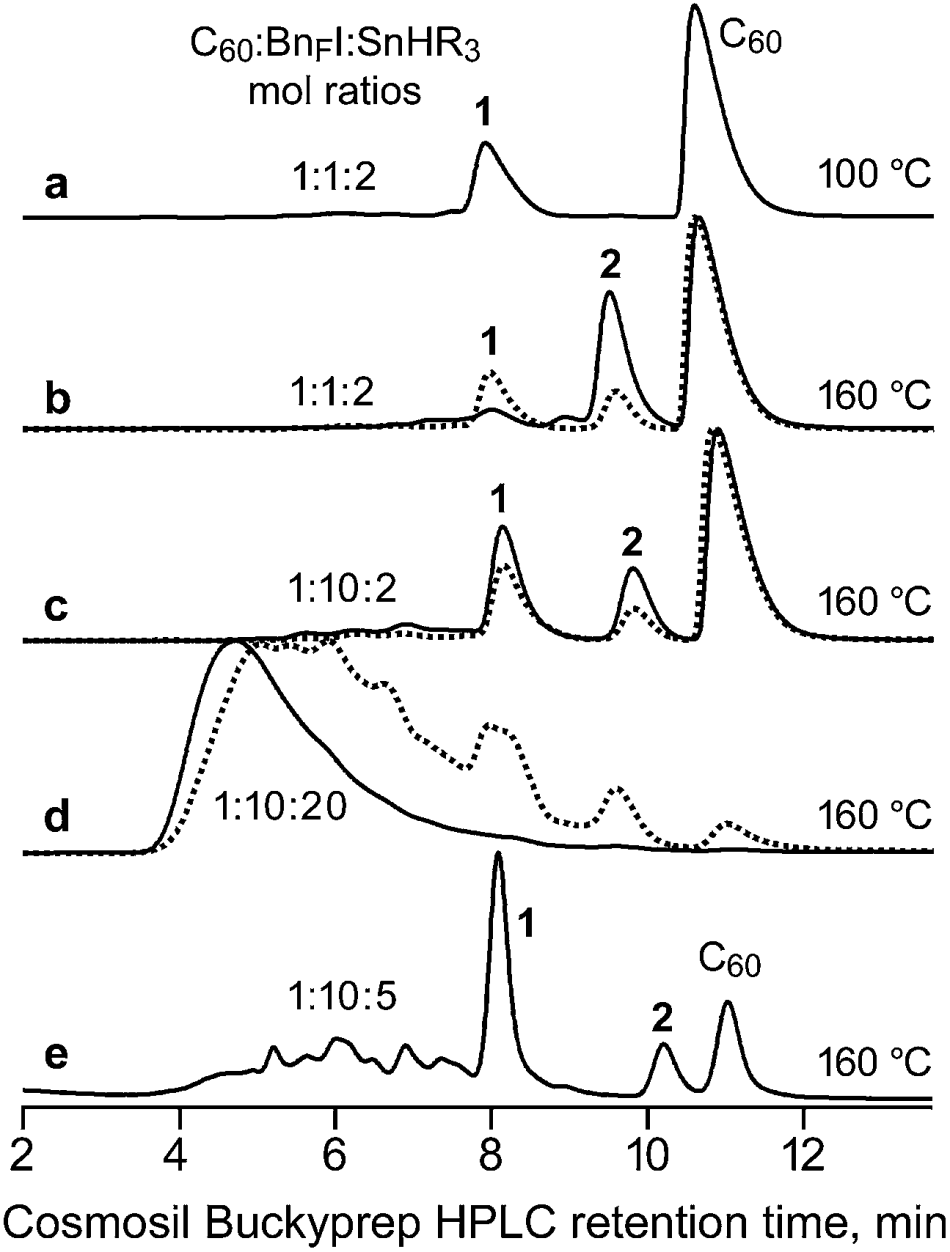
HPLC traces of C_60_ + C_6_F_5_CF_2_I + SnH(*n*-Bu)_3_ reaction mixtures (Bn_F_I = C_6_F_5_CF_2_I; R = *n*-Bu). The dotted-line traces are for 1 h reactions; the solid-line traces are for 2 h reactions. See Table 1 for additional details. Compounds **1** and **2** are 1,9-C_60_(CF_2_C_6_F_5_)H and 1,9-C_60_(*cyclo*-CF_2_(2-C_6_F_4_)), respectively.

In our hands, no AIBN was necessary to prepare **1** when the solvent was 1,2-C_6_H_4_Cl_2_ (oDCB) and the temperature was 160 °C. Furthermore, reaction times of only 1 or 2 h were sufficient to form appreciable amounts of **1**, as shown in Fig. 2 and Table 1. This is probably due to the higher temperature for the reaction and a lower C–I bond energy for C_6_F_5_CF_2_I than for *n*-C_6_F_13_I or *n*-C_12_F_25_I, both of which will result in more C_6_F_5_CF_2_˙ radicals present than the number of R_F_˙ radicals in the reactions of Yoshida *et al.* The mol% values in Table 1 are based on HPLC peak relative integrations and are only approximate. They are listed so that trends in product ratios at various reaction temperatures, reaction times, and reagent mole ratios can be easily understood.

**Table 1:** Reactions producing 1,9-C_60_(CF_2_C_6_F_5_)H (**1**) and 1,9-C_60_(*cyclo*-CF_2_(2-C_6_F_4_)) (**2**)^*a*^

Fig. 2 HPLC trace	Temp., °C	Equiv. Bn_F_I^*b*^	Equiv. SnHR_3_ ^*c*^	Product mixture mol% by HPLC integration^*d*^		
				**1**	**2**	Unreacted C_60_
**a**	100(2)	1	2	24	*ca.* 0	70
**b**	160(5)	1	2	(15)6	(8)30	(70)55
**c**	160(5)	10	2	(18)22	(7)13	(64)51
**d**	160(5)	10	20	(14)3	(5)1	(31) *ca.* 0
**e**	160(5)	10	5	29	7	13

^*a*^All reactions in 1,2-C_6_H_4_Cl_2_ (oDCB). All volatiles (oDCB, I_2_) were removed under vacuum. The solid residue was redissolved in toluene, injected into a COSMOSIL Buckyprep HPLC column, and eluted with 80/20 (v/v) toluene/heptane. The HPLC traces are shown in Fig. 2.

^*b*^Per equiv. C_60_; Bn_F_I = C_6_F_5_CF_2_I.

^*c*^Per equiv. C_60_; R = *n*-Bu.

^*d*^The mol% values in parentheses are for 1 h reactions; all other mol% values are for 2 h reactions. The mol% values do not add up to 100% because other, unidentified fullerene byproducts were also present.

We propose that the formation of **1** from C_6_F_5_CF_2_I and SnH(*n*-Bu)_3_ in oDCB at elevated temperatures is best represented by the following balanced equation (Bn_F_I = C_6_F_5_CF_2_I; R = *n*-Bu):C_60_ + Bn_F_I + SnHR_3_ → 1,9-C_60_(CF_2_C_6_F_5_)H (**1**) + 1/2 I_2_ + 1/2 Sn_2_R_6_

At 100 °C and C_60_ : R_F_I : SnHR_3_ reagent mole ratios of 1 : 1 : 2 (HPLC trace a in Fig. 2), compound **1** and C_60_ were virtually the only fullerene species present in the reaction mixture after 2 h. The same amount of unreacted C_60_ was also present with the same reagent ratios when the temperature was 160 °C for 1 h (HPLC trace b, dotted line), but in this case both **1** and **2** were present (in a *ca.* 2 : 1 mol ratio). After 2 h (trace b, solid line), significantly less **1** and significantly more **2** were present (now in a *ca.* 1 : 5 mol ratio). The HPLC traces labeled c and d show the results of changing the reagent mole ratios for 1 h (dotted lines) and 2 h (solid lines) reactions. HPLC traces d indicate that a large excess of SnHR_3_ produces many other fullerene derivatives (presumably various hydrofullerenes) and much less **1** and **2** than when less SnHR_3_ was used. We conclude that **1** is an intermediate in the formation of **2** under the reaction conditions. It is possible that SnR_3_˙ radicals are involved, as shown in the following *speculative* balanced equation, but SnFR_3_ has not been positively identified:1,9-C_60_(CF_2_C_6_F_5_)H (**1**) + 2SnR_3_˙ → 1,9-C_60_(*cyclo*-CF_2_(2-C_6_F_4_)) (**2**) + SnHR_3_ + SnFR_3_

HPLC trace e in Fig. 2 represents a compromise set of reaction conditions that produced significant amounts of **1** and **2**, relatively less unreacted C_60_, and relatively small amounts of the other fullerene byproducts. This reaction resulted in a 35% isolated yield of **1** and a 7% isolated yield of **2** after HPLC purification (both yields based on C_60_).

An alternate synthesis of **2** is the reaction of **1** with excess Proton Sponge (PS, 1,8-bis(dimethylamino)naphthalene) in CH_2_Cl_2_ at 23(1) °C for 24 h. This reaction, which resulted in a 76% isolated yield of **2** based on **1**, will be discussed in detail in Section 2.3. We also explored photochemical syntheses, but these invariably showed lower yields of **1** and **2** and will not be discussed further.

### 2.2. Characterization of **1** and **2**

The negative-ion (NI) APCI mass spectrum of **1** exhibited an *m*/*z* species at 937, which is consistent with C_60_(CF_2_C_6_F_5_)^–^, or [**1** – H]^–^. The UV-vis spectrum of **1** (Fig. S-2†) exhibited absorption maxima at 324, 431, and 698 nm. The 431 nm band in particular is characteristic of 1,9-C_60_X_2_ or 1,9-C_60_XY derivatives.^17^ In contrast, C_60_XY derivatives with the substituents on the *para* positions on a C_60_ hexagon (*i.e.*, 1,7-C_60_XY) generally exhibit a prominent band at 450 nm.^4^ The singlet at *δ* 7.2 in the ^1^H NMR spectrum of **1** in CDCl_3_ is characteristic of a C_60_–H species^18–20^ (*cf. δ* 6.65 for 1,9-C_60_(CH_2_C_6_H_5_)H^20^).

The NI-APCI mass spectrum of **2** exhibited an *m*/*z* species at 918, which is consistent with the formula C_60_(CF_2_C_6_F_4_)^–^. The UV-vis spectrum of **2** (Fig. S-2†) exhibited bands at 331, 430, and 687 nm, which supports a 1,9-addition pattern for this compound as well (verified by X-ray crystallography). No resonance was observed in a ^1^H NMR spectrum of **2**.

The structure of **2**, determined by X-ray diffraction, is shown in Fig. 1. The five-membered carbocycle substituent is essentially planar, with out-of-plane displacements (OOPs) for C1, C2, C7, C8, and C9 that range from 0.003 Å to 0.066 Å (average ±0.042 Å). In fact, C1, C9, and all seven of the perfluorinated substituent's C atoms are also co-planar (the nine OOPs range from 0.003 to 0.089 Å and average ±0.032 Å). The long C1–C9 bond distance of 1.611(3) Å is typical of C_60_ derivatives with 3-, 4-, 5-, and 6-membered exocyclic rings.^21,22^

The molecule has idealized *C*_s_ symmetry, with the essentially planar faux hawk substituent nearly perpendicular (*i.e.*, 84°) to a plane tangent to the idealized fullerene surface at the C1–C9 midpoint (the two C2–C1–C_cage_ angles only differ by *ca.* 2°; the same is true for the two C8–C9–C_cage_ angles). This gives the molecule its “faux-hawk-hairstyle” appearance, as shown in Fig. S-1.† In the OLYP DFT-optimized structure of **2**, the faux hawk substituent is rigorously planar (except for F1 and F2) and rigorously perpendicular to the C_60_ surface. See Table S-1† for a comparison of relevant interatomic distances and angles for the X-ray and OLYP DFT-optimized structures of **2** and Fig. S-3† for a side-by-side comparison of the two structures. Note that the faux hawk substituent in **2** is attached to the type of C_60_ C–C bond that is common to two hexagons. Table S-1† also includes the distances and angles for the OLYP DFT-optimized structure of the isomer with the faux hawk substituent attached a C_60_ C–C bond that is common to a pentagon and a hexagon, showing that the faux hawk substituent is sterically congruent in both isomers. Nevertheless, the DFT-predicted relative energy of the unobserved alternate isomer is 62 kJ mol^–1^ above the energy of the observed isomer. This difference is, therefore, fullerene based and not faux hawk-substituent based. As indicated above, the faux hawk substituent in the unobserved and observed isomers is attached to a 5,6-pentagon–hexagon and a 6,6-hexagon–hexagon C_60_ edge, respectively. Attachment of substituent atoms to a 5,6-edge of C_60_ introduces two C···C double bonds in pentagons, each of which is predicted to raise the energy of the C_60_ core by 33.5 ± 4.2 kJ mol^–1^.^23^

There are several other examples of C_60_ derivatives with five-membered carbocyclic rings (these are formed by 3 + 2 cycloadditions of trimethylenemethanes to C_60_),^24,25^ but **2** is the only structurally-characterized example in which the carbocycle contains a C···C double bond and is therefore planar. It is also the only example in which the carbocycle is perfluorinated.

**Fig. 3:**
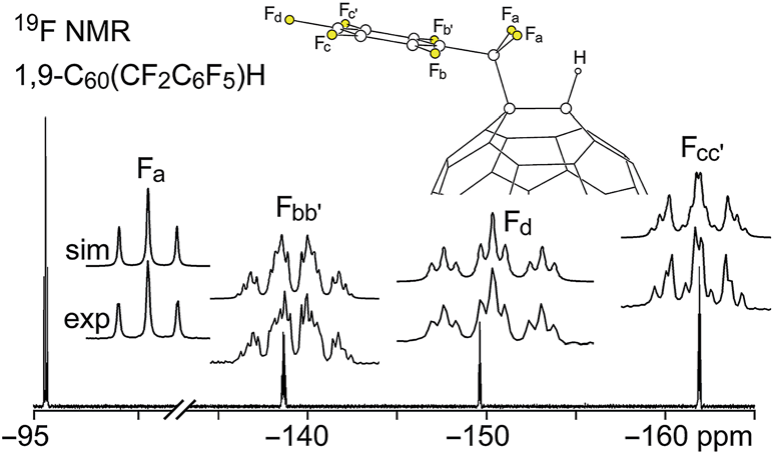
Experimental and simulated 376.5 MHz ^19^F NMR spectra of HPLC-purified 1,9-C_60_(CF_2_C_6_F_5_)H (**1**) in CDCl_3_. Chemical shifts and coupling constants are listed in Table S-2.†

**Fig. 4:**
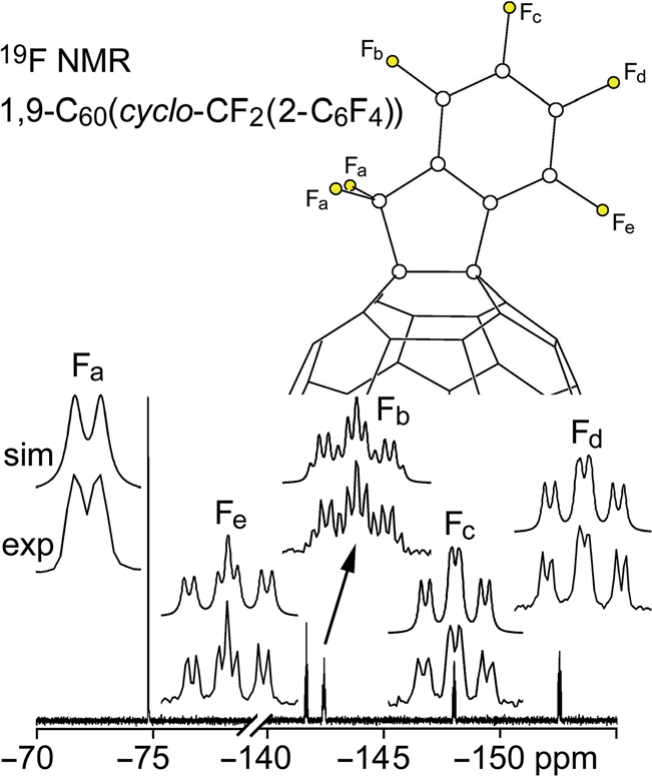
Experimental and simulated 376.5 MHz ^19^F NMR spectra of HPLC-purified 1,9-C_60_(*cyclo*-CF_2_(2-C_6_F_4_)) (**2**) in CDCl_3_. Chemical shifts and coupling constants are listed in Table S-2.†

Fluorine-19 NMR spectra of **1** and **2** are shown in Fig. 3 and 4, respectively. Chemical shifts and coupling constants are listed in Table S-2.† The *J*(FF) coupling constants were determined by simulating the experimental spectra using the program MestReNova 8.1.1. The free rotation about the F_2_C–C_ipso_ bond in **1** and the presumed time-averaged *C*_s_ symmetry of **2** render the F atoms in the CF_2_ moiety magnetically equivalent in both compounds. The aromatic moieties in **1** and **2** exhibited bb′cc′d and bcde patterns, respectively (the notation here conforms to the F atom labels in Fig. 3 and 4).

The *meta* coupling constants *J*(F_b_F_b′_), *J*(F_c_F_c′_), and *J*(F_bb′_F_d_) in **1** and *J*(F_b_F_d_) and *J*(F_c_F_e_) in **2** are all 5–6 Hz. The *ortho* values, *J*(F_b_F_c_)/*J*(F_b′_F_c′_) and *J*(F_cc′_F_d_) in **1** and *J*(F_b_F_c_), *J*(F_c_F_d_), and *J*(F_d_F_e_) in **2**, are, as expected,^26^ significantly larger, 18–26 Hz. The *para* coupling constants, however, are substantially different for the two compounds; *J*(F_b_F_c′_) = *J*(F_b′_F_c_) is 7 Hz in **1** and *J*(F_b_F_e_) is 23 Hz in **2**. The 7 Hz value for **1** is the same as the *ca.* 7 Hz coupling constants for F atoms *para* to one another in perfluorophenyl groups.^27^ The 23 Hz value for **2** can be compared with the 18–26 Hz range for F atoms *para* to one another in tri- and tetrafluorobenzo[*b*]thiophenes,^28^ the 14–19 Hz range in polyfluoroindenes,^29^ and the 12–16 Hz range in tetrafluorobenzo[*b*]thiazoles,^30^ compounds that, like **2**, have a polyfluorobenzo moiety fused to a coplanar five-membered ring. The origin of the difference in magnitude for *para J*(FF) values for polyfluorophenyl *vs.* polyfluorobenzo compounds is not well understood.

On the other hand, the substantial difference in ^4^ *J*(F_a_F_bb′_) in **1** and ^4^ *J*(F_a_F_b_) in **2**, 30 Hz and 5.5 Hz, respectively, has a compelling explanation (the ^4^ *J*(F_a_F_bb′_) value for C_6_F_5_CF_2_I is also 30 Hz). In both cases the F atoms are separated by a C(sp^2^)–C(sp^3^) single bond as well as a C(sp^2^)–C(sp^2^) bond, and the ^4^ *J*(FF) values are almost certainly dominated by Fermi-contact through-space interactions,^31–40^ which are strongly dependent on the F···F distance, the F–C···C(F) angle, and the F–C···C–F torsion angle. The two F_a_···F_b_ distances in the X-ray structure of **2** (these are F1···F6 and F2···F6 in Fig. 1) are 2.998(6) and 3.151(6) Å, respectively, near the limit of *ca.* 3.2 Å for observable Fermi-contact through-space coupling between proximal F atoms (the corresponding distances in the *C*_s_-symmetric DFT-optimized structure of **2** are both 3.088 Å).^31–40^ In contrast, the short F_a_···F_b_ distances in the *C*_s_-symmetric lowest-energy DFT-optimized structure of **1** are both 2.587 Å, a distance which is comparable to the 2.60–2.65 Å F···F distances in compounds previously shown to exhibit ^4^ *J*(FF) values of 19, 25, 27, or 48 Hz depending on the aforementioned angles.^39^

**Fig. 5:**
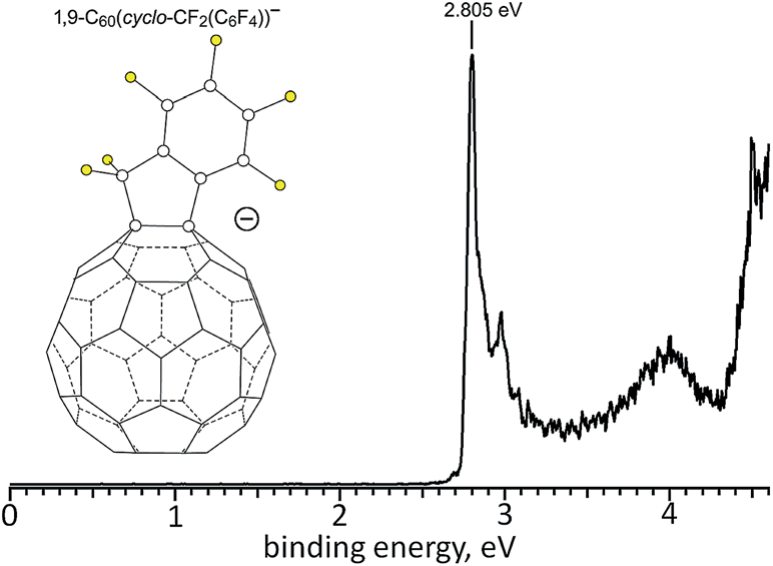
Low-temperature 266 nm photoelectron spectrum of the faux hawk fullerene radical ainon 1,9-C_60_(*cyclo*-CF_2_(2-C_6_F_4_))^–^ (**2** ^–^), from which the 2.805(10) eV gas-phase electron affinity of faux hawk fullerene **2** was determined.

The gas-phase electron affinity (EA) of **2** was determined to be 2.805(10) eV by low-temperature photoelectron spectroscopy (LT-PES) of the **2** ^–^ radical anion, as shown in Fig. 5 (*cf.* 2.683(8) eV for C_60_ (ref. 41) and 2.63(1) eV for PCBM^42^). Therefore, **2** is a stronger electron acceptor (in the gas phase) than C_60_ and PCBM by 0.12(1) and 0.18(1) eV, respectively. The LT-PES spectrum of **1** ^–^ could not be observed because of the rapid loss of the H atom to form the closed-shell species [**1** – H]^–^ (*i.e.*, C_60_(CF_2_C_6_F_5_)^–^). Photodetachment of an electron from this anion allowed the 3.75(3) eV EA of the neutral radical C_60_(CF_2_C_6_F_5_)˙ to be determined, but the EA of **1** remains unknown. It is well known that the EA values for fullerene radicals are *ca.* 1–2 eV higher than for closed-shell fullerene derivatives of similar composition. For example, the EA values for closed-shell C_60_F_46_, the C_60_F_47_˙ radical, and closed-shell C_60_F_48_ are 4.06(25),^43^ 5.66(10),^44^ and 4.06(30) eV,^43^ respectively.

**Fig. 6:**
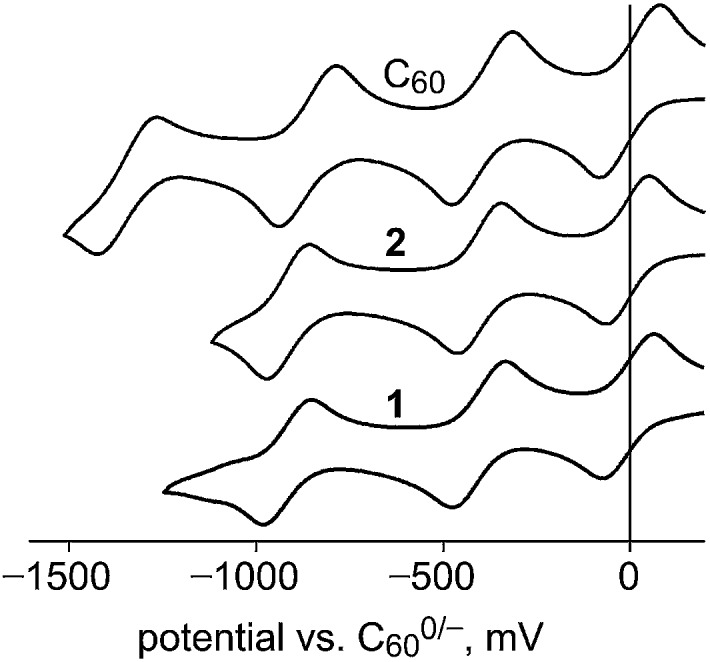
Cyclic voltammograms of C_60_, 1,9-C_60_(CF_2_C_6_F_5_)H (**1**), and 1,9-C_60_(*cyclo*-CF_2_(2-C_6_F_4_)) (**2**) in 1,2-C_6_H_4_Cl_2_ (oDCB) containing 0.1 M N(*n*-Bu)_4_BF_4_ and FeCp_2_ as an internal standard. The FeCp_2_^+/0^ redox waves are not shown.

Square-wave and cyclic voltammograms (SWVs and CVs, respectively) of **1**, **2**, C_60_, and PCBM were recorded under identical conditions in oDCB containing 0.1 M N(*n*-Bu)_4_BF_4_ and Fe(Cp)_2_ as an internal standard. In all cases the reduction potentials determined by SWV and by CV were the same within the ±0.01 V uncertainty of the individual measurements. The potentials are listed in Table 2 along with *E*_1/2_(0/–) values for the related compounds 1,9-C_60_H_2_,^45,46^ 1,9-C_60_(CH_3_)_2_,^47^ and 1,9-C_60_(*cyclo*-C_2_F_4_).^48^ Our *E*_1/2_(0/–) values for C_60_ and PCBM were first reported in 2013 in the same paper reporting the EA of PCBM.^42^ The CVs for **1**, **2**, and C_60_ are shown in Fig. 6. The similarity of *E*_1/2_(0/–) values for **2** and C_60_ is at odds with the 0.12(1) eV difference in their EAs. However, differences in *E*_1/2_(0/–) values for fullerene derivatives are generally smaller, and sometimes much smaller, than the corresponding differences in their EAs.^4,11^

**Table 2:** Electrochemical reduction potentials^*a*^ ^*b*^

Compound	0/– potential, V *vs.* C_60_^0/–^	–/2– potential, V *vs.* C_60_^0/–^	2–/3– potential, V *vs.* C_60_^0/–^	3–/4– potential, V *vs.* C_60_^0/–^
1,9-C_60_(CF_2_C_6_F_5_)H^*b*^	–0.02	–0.45	–0.98	—
1,9-C_60_(*cyclo*-CF_2_(2-C_6_F_4_))^*b*^	–0.01	–0.40	–0.92	–1.36^*c*^
C_60_	0.00	–0.39	–0.85	–1.31
PCBM	–0.09	–0.48	–0.99	
iso-PCBM^*d*^	–0.08			
1,9-C_60_(CH_2_C_6_H_5_)H^*e*^	–0.08	–0.48		
1,9-C_60_H_2_ ^*f*^	–0.13			
1,9-C_60_(CH_3_)_2_ ^*g*^	–0.13			
1,9-C_60_(*cyclo*-C_2_F_4_)^*h*^	0.03			

^*a*^All potentials from cyclic voltammograms unless otherwise indicated. Conditions (unless otherwise noted): purified dinitrogen atmosphere glovebox; 1,2-C_6_H_4_Cl_2_ (oDCB) solutions at 23(1) °C; 0.1 M N(*n*-Bu)_4_BF_4_ electrolyte; Fe(Cp)_2_ internal standard; scan rate 100 mV s^–1^; Pt working and counter electrodes; Ag wire quasi-reference electrode. The uncertainty for each measurement is ±0.01 V.

^*b*^1,9-C_60_(CF_2_C_6_F_5_)H = **1**; 1,9-C_60_(*cyclo*-CF_2_(2-C_6_F_4_)) = **2**.

^*c*^Potential from square-wave voltammetry.

^*d*^ ref. 105.

^*e*^At 25 °C in benzonitrile; ref. 20.

^*f*^At –50 °C in 90/10 (v/v) toluene/dimethylformamide; ref. 46.

^*g*^At 25 °C in benzonitrile; ref. 47.

^*h*^ ref. 48.

Removing one of the double bonds of C_60_ by addition of substituents or a cycloadduct to C1 and C9 generally lowers the *E*_1/2_(0/–) by *ca.* 0.1 V. For example, *E*_1/2_(0/–) values for 1,9-C_60_(CH_2_C_6_H_5_)H,^20^ PCBM,^42^ 1,9-C_60_(CH_3_)_2_,^47^ and 1,9-C_60_H_2_,^46^ are –0.08, –0.09, –0.12, and –0.13 V *vs.* C_60_^0/–^, respectively (in each case the comparison with C_60_ was made under the same conditions of solvent, electrolyte, and temperature). If the cycloadduct is fluorinated and therefore electron withdrawing, as in **2** and 1,9-C_60_(*cyclo*-C_2_F_4_), the *E*_1/2_(0/–) values, –0.01 and 0.03, respectively, have increased by *ca.* 0.1 V from PCBM-like potentials, resulting in C_60_-like potentials. The *E*_1/2_(0/–) values of **2** and C_60_ are the same because the offsetting effects of (i) reducing the fullerene π system by one double bond and (ii) changing the substituent(s) from hydrocarbyl groups or a hydrocarbyl cycloadduct to a perfluorocarbon cycloadduct cancel each other in this case.

The foregoing analysis is the reason that we were surprised that the three *E*_1/2_ values for **1** and **2** are so similar. We expected the *E*_1/2_(0/–) value for **1** to be *ca.* halfway between 0.03 and –0.13 V based on the *E*_1/2_(0/–) values in Table 2, but clearly this is not the case (an ^19^F NMR spectrum of **1** in the electrolyte solution used for the CV experiments, to which 10% C_6_D_6_ was added, verified that **1** does not react with the electrolyte solution on the timescale of the CV experiment). We also expected **1** ^–^ to undergo loss of the H atom to form [**1** – H]^–^, as it did in the LT-PES experiment discussed above and in the **1** + CoCp_2_ reaction discussed below. Furthermore, hydrofullerenes such as 1,9-C_60_H_2_,^45,46^ 1,9-C_60_(CH_2_C_6_H_5_)H,^49^ and isomers of C_70_(CH_2_C_6_H_5_)H^50^ are known to undergo observable H-atom loss upon one-electron reduction unless the CV scan speed is extremely high or the solution is cooled to a low temperature. Nevertheless, our expectations notwithstanding, and in the absence of additional electrochemical experiments, the redox potentials for **1** listed in Table 2 are correctly assigned.

### 2.3. Understanding the transformation **1** → **2** + “HF”

According to O3LYP//OLYP DFT calculations, the transformation **1** → **2** + HF is exothermic by 42 kJ mol^–1^ in the gas phase and 60 kJ mol^–1^ in a PhCN-like dielectric continuum. However, **1** was unchanged after heating an oDCB solution at 160(5) °C for 2 h. Therefore, this reaction does not occur rapidly by a thermally-activated intramolecular pathway in a non-basic solvent. Nevertheless, the synthesis of **1** resulted in the formation of significant amounts of **2** depending on the reaction conditions. To test the idea that **2** can be produced from **1** as an intermediate (although not necessarily as an obligate intermediate), we performed the following series of reactions.

The reagent SnH(*n*-Bu)_3_ and byproduct Sn_2_(*n*-Bu)_6_ that are present during the synthesis of **1** and **2** can form Sn(*n*-Bu)_3_˙ radicals. In a separate experiment, we heated **1** in oDCB at 160 °C with added Sn_2_(*n*-Bu)_6_. Unlike the 160 °C experiment described in the previous paragraph, complete conversion of **1** to **2** occurred within 2 h in the presence of Sn_2_(*n*-Bu)_6_. Since the reagents SnH(*n*-Bu)_3_ and Sn_2_(*n*-Bu)_6_ are not “simple” one-electron reducing agents, we also studied the reaction of **1** with 1 equiv. of CoCp_2_ in PhCN at 23(1) °C. This also caused the conversion of **1** to **2**, as shown by ^19^F NMR spectroscopy.

**Fig. 7:**
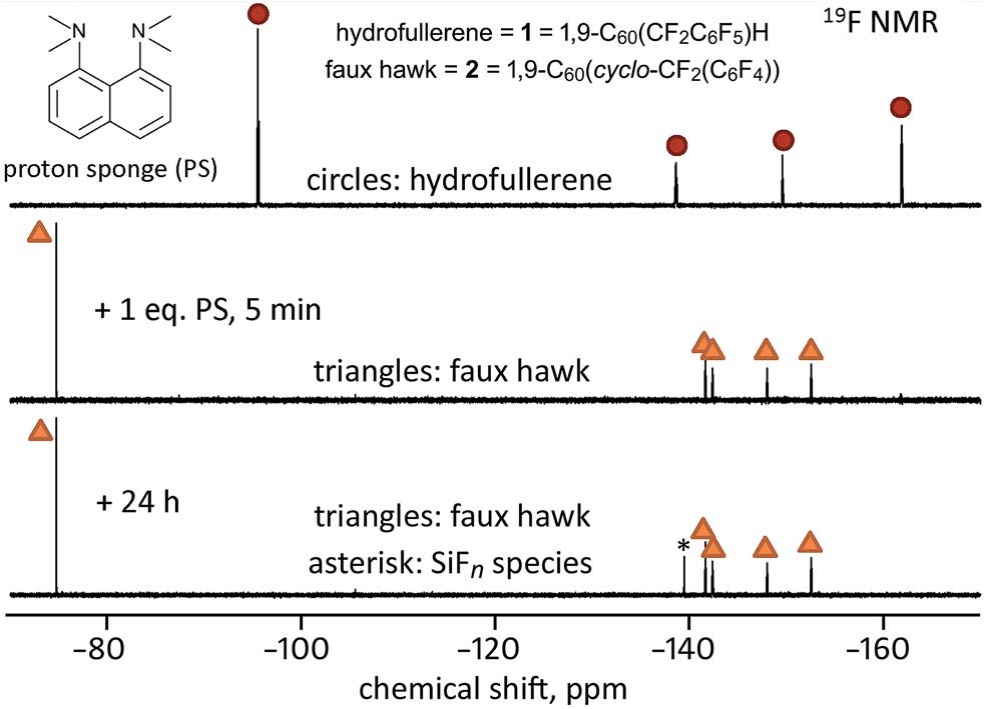
Fluorine-19 NMR spectra (90/10 (v/v) PhCN/C_6_D_6_; 23(1) °C) of the reaction of hydrofullerene **1** with 1.0 equiv. of PS monitored over time. Note that the formation of faux hawk fullerene **2** is complete after only 5 min and that the slow growth of an SiF_*n*_ species (labeled with an asterisk) over 24 h indicates that HF or an HF-like species had been present in solution.

If the one-electron reduced species **1** ^–^ loses an H atom, as do other one-electron reduced hydrofullerenes (see above), the intermediate would be CoCp_2_^+^C_60_(CF_2_C_6_F_5_)^–^ (*i.e.*, CoCp_2_^+^[**1** – H]^–^), which would react further to form **2** and CoCp_2_^+^F^–^. A simpler way to generate [**1** – H]^–^ is by deprotonation. When 1.0 equiv. of the strong base PS was added to a 90/10 (v/v) PhCN/C_6_D_6_ solution of **1** at 23(1) °C, the formation of **2** was complete within 5 min, as shown in Fig. 7. At longer times, a new ^19^F peak appeared at *δ* –139.6. Based on the chemical shift, the magnitude of the coupling constant (145 Hz), and the abundance of the *I* = 1/2 species to which the F atoms are coupled (*ca.* 5%), the new peak is assigned to an “SiF_*n*_” species,^51–56^ indicating that HF, or species with HF-like reactivity towards glass, such as ion-paired [H(PS)]^+^F^–^ and/or HF_2_^–^, were byproducts of the reaction. Rapid exchange between HF, F^–^, and HF_2_^–^ is probably the reason why ^19^F peaks due to one or more of these species were not observed during or after the reaction, only an SiF_*n*_ species due to reaction of the HF-like species with the walls of the NMR tube. When **1** was treated with excess PS in CDCl_3_ for 24 h, the reaction mixture contained 24% **1**, 76% **2**, and a precipitate (the amounts of **1** and **2** were determined by integrating the ^19^F NMR spectrum of the reaction mixture). The precipitate was soluble in CD_3_CN and exhibited a *δ* –155.8 ^19^F NMR singlet and a broad *δ* 19.0 ^1^H NMR singlet, both of which are commensurate with H(PS)^+^F^–^.^57^

**Fig. 8:**
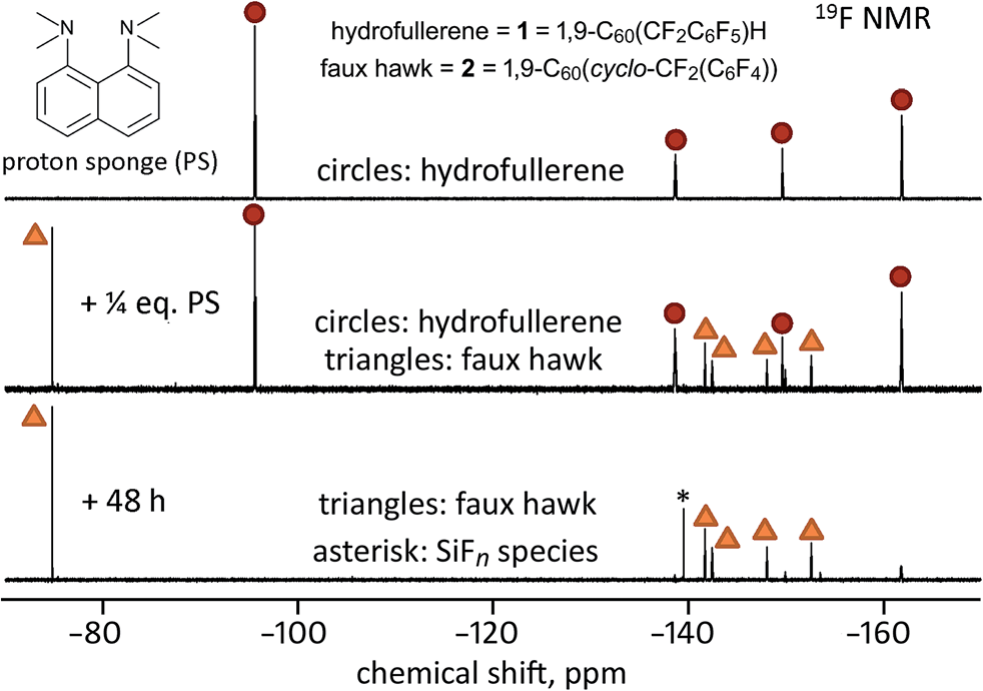
Fluorine-19 NMR spectra (90/10 (v/v) PhCN/C_6_D_6_; 23(1) °C) of the reaction of hydrofullerene **1** with 0.25 equiv. of PS monitored over time. Note that the formation of faux hawk fullerene **2** was not complete within minutes (the middle spectrum) or after 48 h, and that the slow growth of an SiF_*n*_ species (labeled with an asterisk) over 48 h indicates that HF or an HF-like species had been present in solution. Note also that a trace amount of **1** is present in the bottom spectrum.

On the basis of the experiments just described, we propose that the treatment of hydrofullerene **1** with PS resulted in deprotonation to give H(PS)^+^ and C_60_(CF_2_C_6_F_5_)^–^ as first-formed intermediates and that C_60_(CF_2_C_6_F_5_)^–^ formed faux hawk fullerene **2** and “F^–^” within minutes. At longer times, [H(PS)]^+^F^–^ or an equivalent fluoride-like species present reacted with the glass NMR tube to form the SiF_*n*_ species. Even though the putative intermediate C_60_(CF_2_C_6_F_5_)^–^ disappeared too rapidly to observe before an ^19^F NMR spectrum could be recorded, its presence can be proposed because simple deprotonation of hydrofullerenes to give anionic fullerene species is well documented (*i.e.*, hydrofullerenes are known to be Brønsted acids: the p*K*_a_ values for C_60_(CN)H,^58^ C_60_H_2_,^59^ and C_60_(*t*-Bu)H^60^ were found to be 2.5, 4.7, and 5.7, respectively). Interestingly, when **1** was treated with only 0.25 equiv. of PS in 90/10 (v/v) PhCN/C_6_D_6_ solution, the complete conversion to **2** also occurred, but only after 48 h, as shown in Fig. 8. This autocatalytic transformation of **1** into **2** presumably results from the first-formed 0.25 equiv. byproduct F^–^ (or [H(PS)]^+^F^–^ or HF_2_^–^), which formed rapidly, acting as a base and continuing to deprotonate, albeit more slowly, additional molecules of **1** until it is completely converted to **2**. In a control experiment to inhibit the proposed catalytic effect of F^–^ as a general base, a few drops of saturated aqueous Ca(NO_3_)_2_ were added to a similar NMR-scale reaction containing *ca.* 0.3 equiv. of PS (based on **1**). In this case, the conversion of **1** to **2** was only 30–40% complete after 48 h, a white gelatinous precipitate formed in the aqueous layer (presumably CaF_2_), and the ^19^F NMR peak assigned to the SiF_*n*_ species was absent even after 48 h.

The rapid conversion of deprotonated **1** (*i.e.*, [**1** – H]^–^) to **2** most likely occurs by an intramolecular S_N_Ar mechanism whereby the [**1** – H]^–^ fullerene carbanion attacks one of the *ortho*-C–F bonds of the CF_2_C_6_F_5_ substituent. Fullerenes aside, intermolecular S_N_Ar reactions involving aromatic C–halogen bonds have been extensively studied.^61–65^ In contrast, the scope of intramolecular S_N_Ar reactions that result in breaking an aromatic C–F bond and concomitant loss of F^–^ is limited.^66–68^ In the examples most relevant to this work, Hughes and co-workers showed that perfluorobenzyl ligands on either Co^67^ or Rh^68^ can undergo intramolecular S_N_Ar substitution of an *ortho*-F atom to form either six- or five-membered chelate rings, respectively. There is general agreement that, all other things being equal, aromatic C–F bonds undergo S_N_Ar substitution much faster than aromatic C–Cl, C–Br, or C–I bonds.^61–65^ However, there is still controversy about whether a true Meisenheimer^69^ intermediate is formed (even if it cannot be detected spectroscopically)^70–76^ or whether the reaction involves a single Meisenheimer-like transition state.^77–80^

**Fig. 9:**
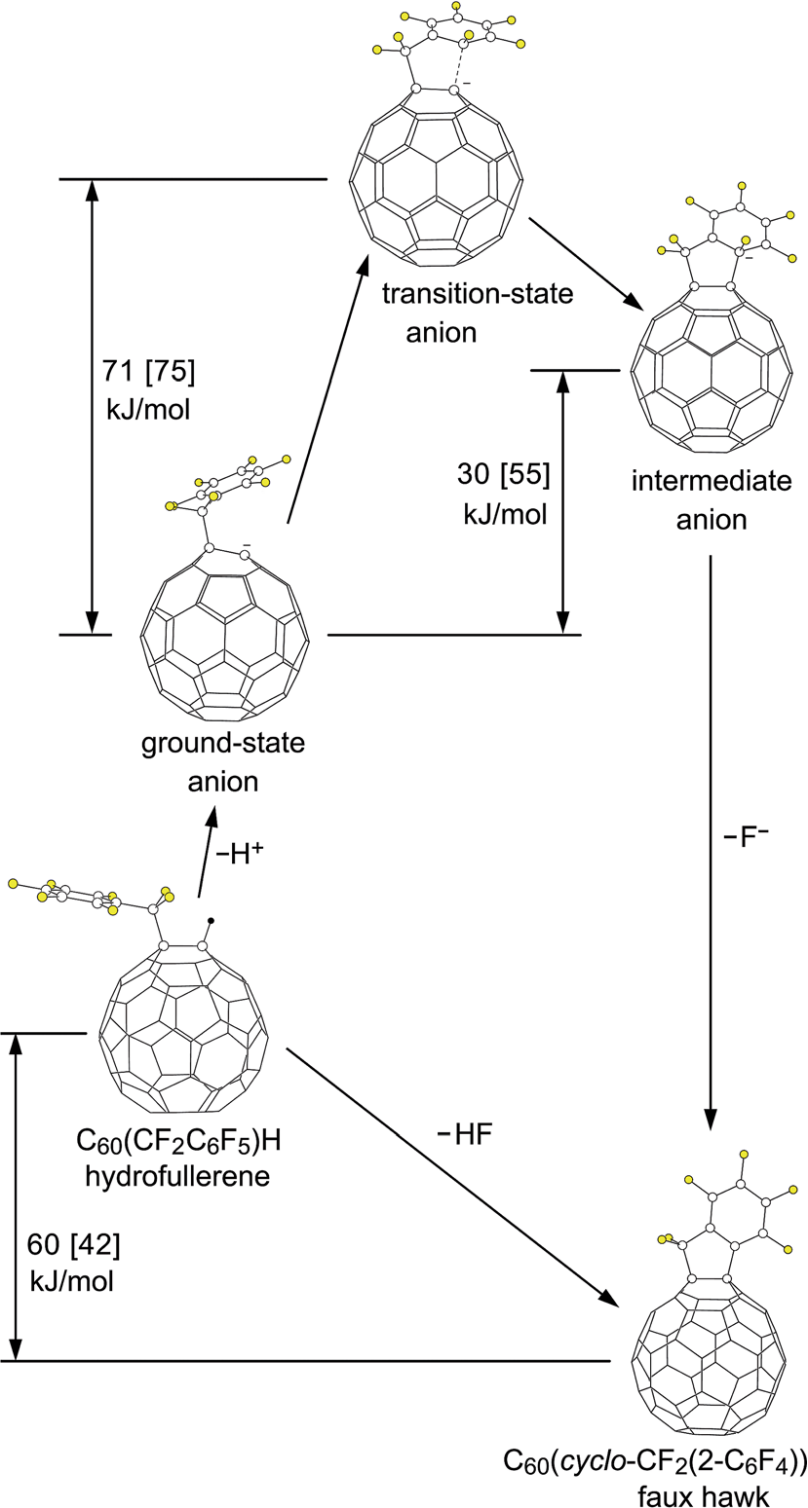
OLYP DFT-optimized structures and O3LYP//OLYP relative energies of 1,9-C_60_(CF_2_C_6_F_5_)H (**1**; hydrofullerene), 1,9-C_60_(*cyclo*-CF_2_(2-C_6_F_4_)) (**2**; faux hawk), and the three [**1** – H]^–^ anions proposed for the S_N_Ar transformation [**1** – H]^–^ → **2** + F^–^ (the ground-state, transition-state, and intermediate C_60_(CF_2_C_6_F_5_)^–^ anions). The energy changes shown, which are not to scale on the vertical axis, are for (i) a dielectric continuum equivalent to benzonitrile (no brackets) and (ii) the gas phase (square brackets).

**Fig. 10:**
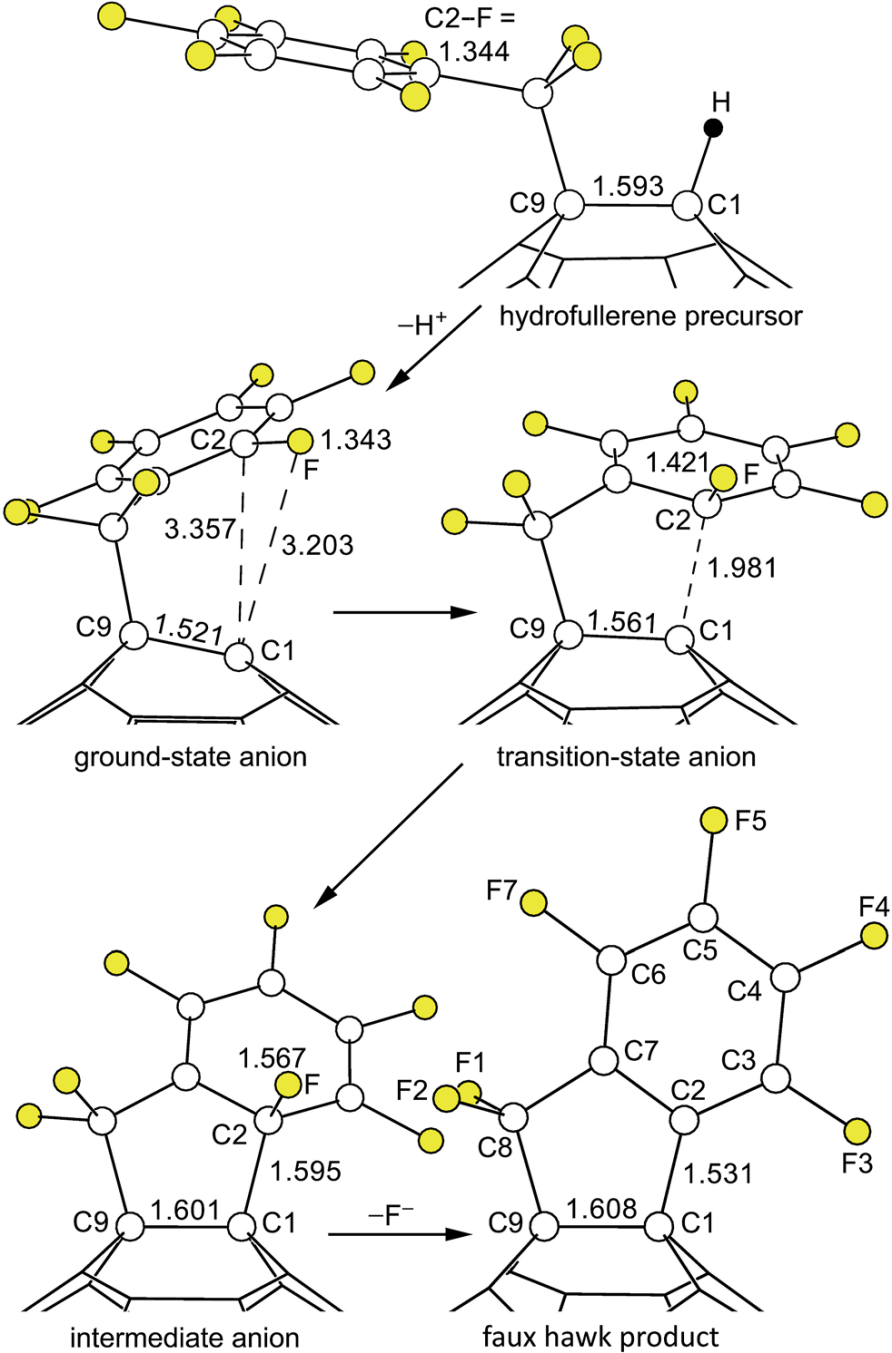
Parts of the OLYP DFT-optimized structures and O3LYP//OLYP relative energies of 1,9-C_60_(CF_2_C_6_F_5_)H (**1**; hydrofullerene), 1,9-C_60_(*cyclo*-CF_2_(2-C_6_F_4_)) (**2**; faux hawk), and the three [**1** – H]^–^ anions proposed for the S_N_Ar transformation [**1** – H]^–^ → **2** + F^–^ (*i.e.*, the ground-state, transition-state, and intermediate C_60_(CF_2_C_6_F_5_)^–^ anions). Additional distances and angles are listed in Table 3 and are shown in Fig. S-7 through S-12.†

Reactions of C_60_R^–^ carbanions with electrophilic substrates EX to form new C_60_(E)R species and X^–^ are well known,^22,81,82^ but to our knowledge there is no previous example of an S_N_Ar reaction involving a fullerene *cage* carbanion (*i.e.*, not including examples such as the negatively-charged N atom of a deprotonated *cyclo*-pyrrolidinofullerene undergoing an intermolecular S_N_Ar reaction with an aryl chloride^83^), let alone an *intra*molecular S_N_Ar reaction of a fullerene cage carbanion attacking an Ar–F bond. Therefore, we decided to test the intramolecular S_N_Ar hypothesis for the observed transformation [**1** – H]^–^ → **2** + F^–^ by determining DFT-optimized structures and relative energies for **1** and **2** as well as for three different states of [**1** – H]^–^. Fig. 9 shows the OLYP DFT-optimized structures and the O3LYP//OLYP relative energies of these five species. Both gas-phase and PhCN-like dielectric continuum relative energies were calculated. Drawings of the upper fragments of the gas-phase optimized structures are shown in Fig. 10 and relevant interatomic distances and angles are listed in Table 3.^84^ Larger drawings of the optimized species are shown in Fig. S-7 to S-12.† The calculated solvation energies for the ground-state (GS), transition-state (TS), and Meisenheimer-like intermediate-state (IS) structures of the deprotonated [**1** – H]^–^ anion are listed in Table S-3.† This table also lists the gas-phase relative energies using other DFT functionals for the three [**1** – H]^–^ states along the proposed S_N_Ar reaction coordinate.

**Table 3:** DFT-predicted interatomic distances (Å) and angles (deg) for species along the proposed S_N_Ar reaction coordinate leading from **1** to **2** + HF^*a*^

Distance or angle	**1**	Ground-state [**1** – H]^–^ anion	Transition state [**1** – H]^–^ anion	Intermediate [**1** – H]^–^ anion	**2** ^*b*^
C1–C2	—	3.357	1.981	1.595	1.531
C1–C9	1.593	1.521	1.561	1.601	1.608
Other C1–C_cage_	1.527, 1.527	1.424, 1.425	1.474, 1.484	1.528, 1.533	1.541, 1.541
C2–F	1.344	1.343	1.421	1.567	—
Other C_Ar_–F	1.342 × 2, 1.339, 1.344	1.345 × 2, 1.347 × 2	1.354, 1.356, 1,358, 1.359	1.356, 1.357, 1.360, 1.364	1.340, 1.341, 1.342, 1.348
C2–C3	1.397	1.398	1.431	1.456	1.395
C2–C7	1.406	1.406	1.444	1.457	1.395
Other C_Ar_–C_Ar_ ^*c*^	1.395 × 2, 1.397, 1.406	1.393 × 2, 1.397, 1.405	1.380, 1.407, 1.392, 1.396	1.375, 1.390, 1.392, 1.413	1.395, 1.396, 1.399, 1.401
C1–C2–C3	—	136.3	117.1	120.1	128.1
C1–C2–C7	—	67.6	98.2	106.2	112.9
C3–C2–C7	121.8	121.7	116.5	113.3	119.0
F–C2–C1	—	71.9	94.9	101.0	—
F–C2–C3	116.5	116.1	111.6	105.6	—
F–C2–C7	121.7	122.2	116.1	109.7	—
C1 POAV *θ*_p_ ^*d*^	18.2	9.6	16.2	19.6	19.3
C9 POAV *θ*_p_ ^*d*^	19.5	22.0	20.0	19.0	19.1

^*a*^OLYP DFT-optimized structures. **1** = 1,9-C_60_(CF_2_C_6_F_5_)H; **2** = 1,9-C_60_(*cyclo*-CF_2_(2-C_6_F_4_)).

^*b*^A comparison of the DFT-predicted and experimental X-ray diffraction distances and angles for **2** is shown in Table S-1.

^*c*^These four distances are listed in the order C3–C4, C4–C5, C5–C6, and C6–C7.

^*d*^The π-orbital axis vector (POAV) for a fullerene C atom is defined as the vector that makes equal angles to the three C_cage_ atoms to which it is attached (see ref. 84). The common angle is denoted *θ*_σπ_ and *θ*_p_ = *θ*_σπ_ – 90°. The angle *θ*_p_ denotes the degree of pyramidalization of a fullerene cage C atom. For an idealized trigonal-planar C(sp^2^) atom, *θ*_p_ = 0°; for an idealized tetrahedral C(sp^3^) atom, *θ*_p_ = 19.5°.

The DFT results show that an S_N_Ar mechanism is energetically viable for the unimolecular intramolecular annulation reaction [**1** – H]^–^ → **2** + F^–^, even without the probable stabilizing effect of hydrogen bonding of either H(PS)^+^ or HF to the three [**1** – H]^–^ structures. The transition state structure of [**1** – H]^–^ is only *ca.* 70 kJ mol^–1^ above the ground-state structure; transition states of 45–130 kJ mol^–1^ have been calculated for non-fullerene S_N_Ar transition states involving nitrogen or sulfur nucleophiles and aromatic C–F bonds.^74–76^ This is consistent with the observed reaction time of only minutes when **1** was mixed with 1 equiv. of PS in 90/10 (v/v) PhCN/C_6_D_6_ at 23(1) °C. Apparently, there is sufficient conformational flexibility in the CF_2_C_6_F_5_ substituent in [**1** – H]^–^ to accommodate the nascent five-membered ring in the transition state.

The structural changes in the C1–C9 moiety of five fullerene species along the proposed S_N_Ar reaction coordinate can be appreciated using Fig. 10 and the results listed in Table 3. There is a significant change in the degree of pyramidalization (*θ*_p_; see Table 3) of C1 and in the set of three C1–C distances for the first step in the reaction sequence, the deprotonation of **1**. The former changes from 18.2° for **1** to 9.6° for GS [**1** – H]^–^ and the latter from {1.59, 1.53, 1.53 Å} for **1** to {1.52, 1.42, 1.43} for GS [**1** – H]^–^, signaling a change in hybridization of C1 from sp^3^ in **1** to a blend of sp^3^ and sp^2^ in GS [**1** – H]^–^. The ground-state anion is a carbanion, but the negative charge and the putative “lone pair” are delocalized throughout the C_60_ cage. Significantly, the 9.6° *θ*_p_ degree of pyramidalization for C1 in GS [**1** – H]^–^ is smaller, not larger, than the 11.6° *θ*_p_ value for the cage C atoms in C_60_ (ref. 84) (the delocalization of the negative charge in C_60_R^–^ carbanions was previously proposed by Van Lier, Geerlings, and coworkers based on computational results^85–87^). As expected, the C_6_F_5_ rings in **1** and GS [**1** – H]^–^ are virtually congruent. Even the C8–C9 bond distance is unaffected by the deprotonation.

In the second step, GS [**1** – H]^–^ is transformed into TS [**1** – H]^–^. Even though the C1···C2 distance, at 1.981 Å, is very long, the C1 *θ*_p_ value increases from 9.6° to 16.2°, which is 90% of its original value in **1**. Accordingly, the three C1–C_cage_ distances increase from {1.52, 1.42, 1.43} in GS [**1** – H]^–^ to {1.56, 1.47, 1.48} in TS [**1** – H]^–^. At the same time, C2 is developing sp^3^ character: the C2–C3 and C2–C7 distances increase from 1.40 and 1.41 Å in GS [**1** – H]^–^ to 1.43 and 1.44 Å in TS [**1** – H]^–^, and the sum of the three angles at C2 involving C3, C7, and F is 344° in TS [**1** – H]^–^ whereas this sum is 360° in GS [**1** – H]^–^. Another way to depict the distortion in the C_6_F_5_ group in TS [**1** – H]^–^ is as follows. The 10 atoms C2–C7 and F3–F6 are coplanar to within ±0.02 Å in both GS [**1** – H]^–^ and TS [**1** – H]^–^. However, in GS [**1** – H]^–^ atom F (*i.e.*, the F atom bonded to C2) is also in that plane whereas in TS [**1** – H]^–^ it is displaced 0.86 Å from that plane. As expected, the C2–F bond in TS [**1** – H]^–^, at 1.42 Å, is significantly longer than the 1.34 Å distance in both hydrofullerene precursor **1** and the GS [**1** – H]^–^ anion.

The Meisenheimer-like intermediate, denoted IS [**1** – H]^–^, exhibits further repyramidalization of C1 and further pyramidalization of C2. Both of these atoms are essentially tetrahedral in the intermediate, with four single bonds. In fact, the C1 *θ*_p_ value, 19.6°, is only 0.1° different than the ideal *θ*_p_ tetrahedral angle (19.5°), and the sum of the three angles at C2 involving C3, C7, and F is 328.6°, within 0.1° of the expected sum for a tetrahedral C atom (*i.e.*, 3 × 109.5° = 328.5°). Furthermore, the C2–F bond, at 1.567 Å, is exceptionally long and is clearly developing a significant amount of F^–^ character. Note that all C–F bond distances measured by X-ray crystallography (as of 1987) are shorter than 1.4 Å.^88^

Finally, in the last step of the reaction sequence shown in Fig. 9 and 10, F^–^ dissociates from the intermediate and the C_6_F_4_ ring undergoes rearomatization (*i.e.*, the C2–C3 and C2–C7 bond distances shorten from 1.46 Å in IS [**1** – H]^–^ to 1.40 Å in **2** (therefore all six C_Ar_–C_Ar_ distances in **2** are 1.40 Å)).

### 2.4. Molecular structure and solid-state packing of **2** and comparison with single-crystal X-ray structures of PCBM

There are two solvent-free X-ray structures of PCBM: a single-crystal structure determined using data collected at 100(2) K (ref. 89) and a structure determined by powder X-ray diffraction data collected at 298(2) K.^90^ The molecular structures of **2** and the 100 K single-crystal structure PCBM^89^ are shown side-by-side in Fig. S-13.† The two substituents have nearly the same number of non-hydrogen atoms, 13 for **2** and 14 for PCBM, but the faux hawk substituent is clearly the more compact. The 1.632(2) Å C1–C9 bond in PCBM is only marginally longer than the 1.610(5) Å distance in **2**, and fullerene cage atoms C1 and C9 are only slightly less pyramidalized in PCBM (POAV *θ*_p_ = 17.1° × 2) than in **2** (*θ*_p_ = 18.9 and 19.1°).

The solvent-free solid-state packing of **2** and PCBM^89^ are analyzed in detail and discussed in the ESI,† along with comparisons to PCBM X-ray structures containing solvent molecules and a related structure (see page S-17 and Fig. S-14 through S-18†). The result of this analysis is that there are only seven (7) nearest neighbor fullerene molecules in crystalline solvent-free PCBM, with C_60_ centroid···centroid (⊙···⊙) distances of 9.95–10.28 Å. The mean distance is 10.17 Å. On the other hand, there are ten (10) nearest neighbors in the structure of **2**, with ⊙···⊙ distances of 9.74–10.34 Å. The mean distance is 10.09 Å. The result is that the density of crystalline **2**, 1.885 g cm^–3^, is 15.6% higher than the 1.631 g cm^–3^ density of solvent-free PCBM, even though the molar masses of the two compounds, 918.67 g mol^–1^ for **2** and 910.83 g mol^–1^ for PCBM, differ by only 1.1%. The significance of this is that the aggregation behavior of OPV acceptor fullerenes in the solid state, especially the number of electronically coupled nearest neighbors and their three-dimensional arrangement, is widely believed to be among the key factors that determine charge transport properties in the fullerene domains in Type II heterojunction solar cells.^89–100^

### 2.5. Microwave conductivity experiments

**Fig. 11:**
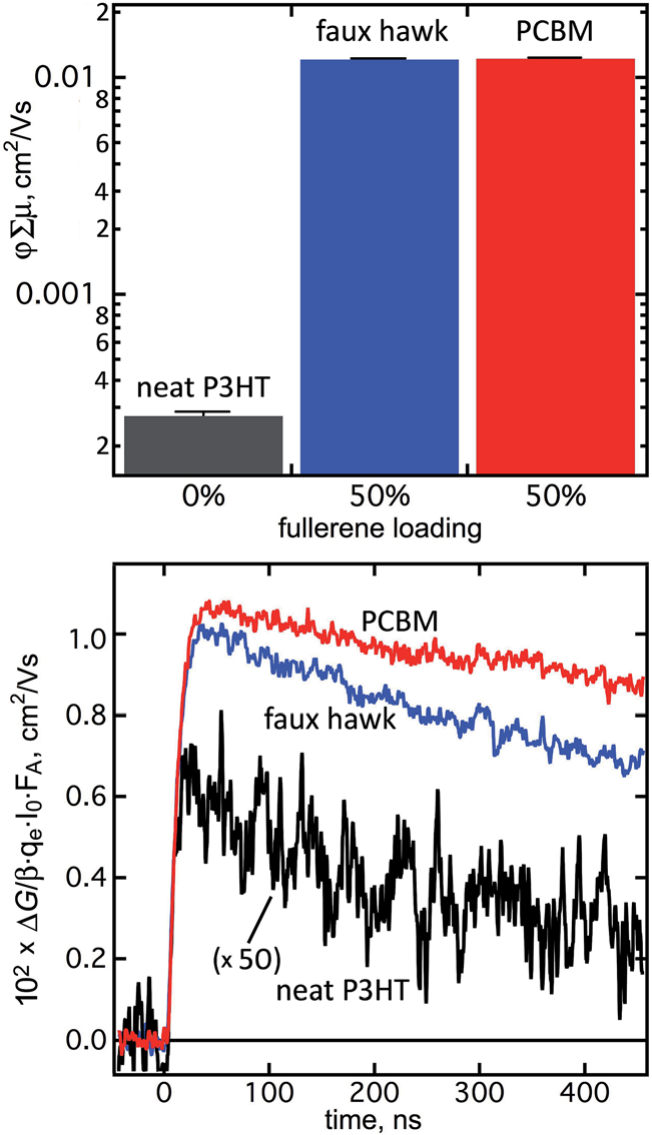
(Top) Peak *φ*∑*μ* values for thin films of neat P3HT and 50/50 (wt/wt) blends of P3HT and either faux hawk fullerene 1,9-C_60_(*cyclo*-CF_2_(2-C_6_F_4_)) (**2**) or PCBM. The uncertainty for each measurement is shown on each bar. (Bottom) Transient profile decay curves over 450 ns at incident 500 nm photon fluxes of *ca.* 1 × 10^13^ cm^–2^ for neat P3HT and *ca.* 2 × 10^11^ cm^–2^ for the blends (*ΔG* is the change in photoconductance, *β* is the ratio of the waveguide cross-section dimensions (2.2 in the instrument used), *q*_e_ is the electron charge, *I*_0_ is the incident photon flux, and *F*_A_ is the fraction of photons absorbed by the sample).

The denser packing of **2** relative to PCBM and the nearly-equal *E*_1/2_(0/–) values for **2** and C_60_ suggested that **2** might be an efficacious electron acceptor in OPV bulk heterojunction thin films. To test this hypothesis, we probed the charge generation and decay dynamics of **2** when blended with regioregular poly-3-hexylthiophene (rr-P3HT) using time-resolved microwave conductivity (TRMC).^101^ There are two advantages to measuring photoconductance with TRMC: (i) it is a contactless method and is therefore specific to processes occurring in an OPV active-layer film under illumination; and (ii) the ns–μs timescale of TRMC measurements is the same as the timescale of charge-carrier dynamics in an OPV device.^95^ Fig. 11 shows the *φ*∑*μ* TRMC figure of merit for three thin-film samples (*φ* is the quantum yield of mobile-charge-carrier generation (*i.e.*, electrons and holes) and ∑*μ* is the sum of charge-carrier mobilities at the limit of low excitation intensity).^102^

The *φ*∑*μ* value for a blend of rr-P3HT and **2** is nearly two orders of magnitude higher than for a neat rr-P3HT thin film and is comparable to the *φ*∑*μ* value for an rr-P3HT/PCBM blend, as shown in Fig. 11. The latter observation is indicative of efficient free-charge-carrier generation in the rr-P3HT/**2** blend, a combination of a high *φ* value as well as a large ∑*μ* contribution due to electron mobility in domains of **2** within the bulk heterojunction thin film, as previously observed for rr-P3HT blends with other high-performance OPV acceptors.^95,102,103^

The decay profiles of the transients for the rr-P3HT/**2** and rr-P3HT/PCBM blends are nearly identical, as also shown in Fig. 11. The signals are longer lived than for the neat donor polymer, which is normally attributed to high electron mobility in the fullerene phase.^102^ Taken together, the TRMC results indicate that **2** is a promising acceptor for OPV. Its higher electron affinity relative to PCBM suggests that it may be better to blend **2** with “push–pull” low-bandgap donor polymers with HOMO and LUMO energies deeper than P3HT in order to offset open-circuit-voltage losses,^104^ and the perfluorinated nature of its substituent suggests and it may be better to blend **2** with fluorinated donor polymers. These experiments are currently underway and will be reported in a future publication.

### 2.6. Thermal stability of 1,9-C_60_(*cyclo*-CF_2_(2-C_6_F_4_)) (**2**)

**Fig. 12:**
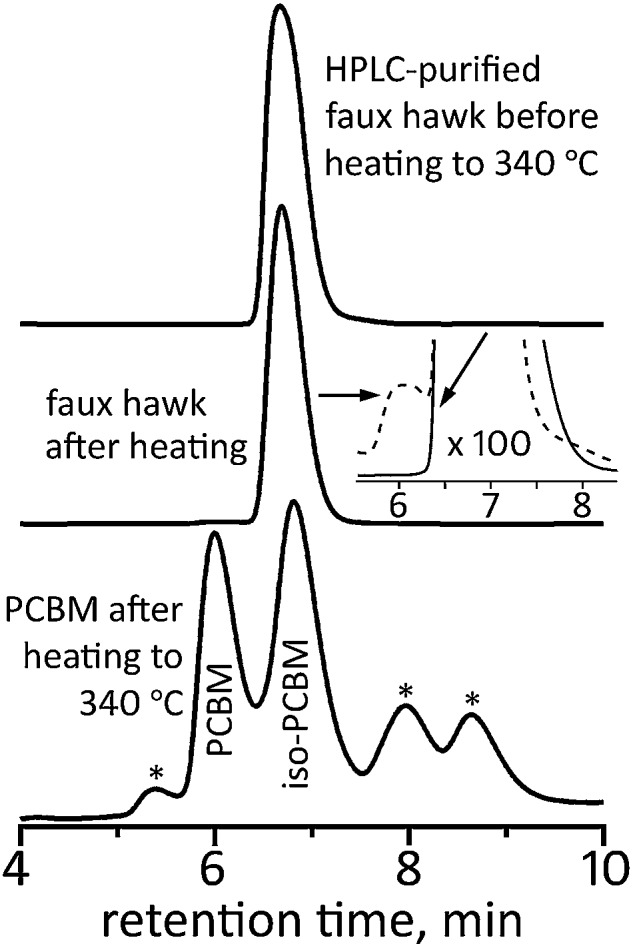
HPLC traces of faux hawk fullerene 1,9-C_60_(*cyclo*-CF_2_(2-C_6_F_4_)) (**2**) before and after heating to 340 °C for 20 min and HPLC trace of PCBM after heating to 340 °C for 20 min. The PCBM HPLC data were reported in ref. 105. The inset for the middle HPLC trace has been expanded 100 times on the vertical axis (the after-heating trace in the inset is the dashed line). The asterisks in the PCBM after-heating trace are unidentified thermal decomposition products. For all three HPLC traces, a COSMOSIL Buckyprep column was used with a toluene eluent rate of 5 mL min^–1^ and 300 nm detection.

The final comparison we wish to report is the thermal stability of **2** *vs.* PCBM. It was recently shown that PCBM undergoes substantial decomposition in only 20 min at 340 °C.^105^ An HPLC trace of 340 °C-treated PCBM, taken from a figure in ref. 105, is shown in Fig. 12. Part of the 340 °C-treated PCBM sample was a charred residue that did not dissolve in toluene. Of the portion of the sample that did dissolve, only *ca.* 22% was intact PCBM. The most abundant decomposition product was identified as a new five-membered ring cycloadduct isomer of PCBM that was named iso-PCBM and that is virtually a hydrocarbyl equivalent of **2** (see Fig. S-19† for the structure of iso-PCBM; see also Table 2).^105^

In contrast, the HPLC trace of 340 °C-treated **2**, also shown in Fig. 12, shows no evidence of decomposition unless the traces are vertically expanded 100 times. In the expanded trace, the unambiguous presence of one yet-unidentified new species with an abundance of *ca.* 0.6 mol% based on HPLC relative intensities can be seen. In addition, no new peaks were observed in the ^19^F NMR spectrum of 340 °C-treated **2**. Based on the signal/noise ratio of that spectrum, the upper limit of any fluorine-containing compound other than **2** is *ca.* 0.5 mol%. Significantly, there was no insoluble residue after **2** was heated at 340 °C.

These results are important because post-fabrication thermal annealing of fullerene-containing OPV devices can, in some cases, improve device efficiency and therefore have become common practice in OPV research^106,107^ and because thin films of PCBM or similar fullerene derivatives used for photophysical or electronic property investigations were prepared by high-temperature vacuum sublimation^108–110^ (see also additional references cited in ref. 105). It is possible that the thin films and other materials/devices studied in the papers just cited contained iso-PCBM as well as PCBM and possibly other PCBM thermal decomposition products. How well faux hawk fullerene **2** performs not only in OPV but in other organic electronic applications, especially those that involve thermal annealing and/or thermal evaporation at temperatures up to and including 340 °C, remains to be seen.

## 3. Experimental section

### 3.1. General methods, reagents, and solvents

An inert-atmosphere glovebox and/or standard benchtop inert-atmosphere techniques^111^ (dioxygen and water vapor levels ≤1 ppm) were used to perform reactions and, in general, to prepare samples for spectroscopic, electrochemical, and microwave conductivity analysis. Following filtration through silica gel, reaction mixtures were exposed to air, in most cases with minimal exposure to light. HPLC purifications were also performed in the presence of air.

The following reagents and solvents were obtained from the indicated sources and were used as received or were purified/treated/stored as indicated: C_60_ (MTR Ltd., 99.5+%); phenyl-C_61_-butyric acid methyl ester (PCBM, Nano-C, 99+%); regioregular (rr) poly-3-hexylthiophene (rr-P3HT, Sigma-Aldrich, 90+% rr); heptafluorobenzyl iodide (C_6_F_5_CF_2_I, SynQuest, 90%); tri-*n*-butyltin hydride (SnH(*n*-Bu)_3_, Strem Chemicals, 95+%), hexabutylditin(Sn–Sn) (Sn_2_(*n*-Bu)_6_, Alfa Aesar, 98%); 1,2-dichlorobenzene (oDCB, Acros Organics, 99%, dried over and distilled from CaH_2_); dichloromethane (DCM, Fisher Scientific, ACS grade); benzonitrile (PhCN, Aldrich, 99+%, dried over 3 Å molecular sieves); chloroform-d (CDCl_3_, Cambridge Isotope Labs, 99.8%); benzene-d_6_ (C_6_D_6_, Cambridge Isotope Labs, dried over 3 Å molecular sieves), hexafluorobenzene (Oakwood Products); 1,4-bis(trifluoromethyl)benzene (C_8_H_4_F_6_, Central Glass Co., 99%); ferrocene (FeCp_2_, Acros Organics, 98%); cobaltocene (CoCp_2_, Strem Chemical, purified by sublimation and stored in the glovebox); silica gel (Sigma-Aldrich, 70–230 mesh, 60 Å); 1,8-bis(dimethylamino)naphthalene (Proton Sponge (PS), C_14_H_18_N_2_, Sigma-Aldrich, purified by sublimation and stored in the glovebox); toluene (Fisher Scientific, ACS grade); heptane (Mallinckrodt, ACS grade); acetonitrile (Mallinckrodt Chemicals, ACS grade); and tetra-*n*-butylammonium tetrafluoroborate (N(*n*-Bu)_4_BF_4_, TBABF_4_, Fluka, puriss grade, dried under vacuum at 70 °C for 24 h and stored in the glovebox).

### 3.2. Synthesis of compounds

#### 1,9-C_60_(CF_2_C_6_F_5_)H

The compounds C_60_ (120 mg, 0.167 mmol), C_6_F_5_CF_2_I (0.263 mL, 1.67 mmol), and SnH(*n*-Bu)_3_ (0.225 mL, 0.835 mmol) were dissolved in oDCB, heated at 160(5) °C for 2 h, and cooled to 23(1) °C. All volatiles, including the byproduct I_2_, were removed from the purple reaction mixture under vacuum. The solid residue was dissolved in toluene, added to a preparative-scale COSMOSIL Buckyprep HPLC column by injection (see below), and eluted with 80/20 (v/v) toluene/heptane at 16 mL min^–1^ (the HPLC trace is shown in Fig. 2e). The fraction that eluted from 8.0 to 8.3 min was collected and evaporated to dryness under vacuum, yielding 55 mg of **1** (35% yield based on C_60_). The ^19^F NMR spectrum of the isolated product (Fig. 3) demonstrates that compound **1** prepared in this way is at least 97 mol% pure.

#### 1,9-C_60_(*cyclo*-CF_2_(2-C_6_F_4_))

The fraction of the HPLC purification described above that eluted between 9.9 and 10.6 minutes was collected and evaporated to dryness, yielding 11 mg of **2** (7% yield based on C_60_). The ^19^F NMR spectrum of the isolated product (Fig. 4) demonstrates that compound **2** prepared in this way is at least 95 mol% pure.

Alternatively, **1** (5.0 mg) was treated with excess Proton Sponge (PS) in CH_2_Cl_2_ at 23(1) C for 24 h. The brown reaction mixture was filtered through silica gel to remove [H(PS)]^+^F^–^ and unreacted PS. The filtrate was evaporated to dryness under vacuum. The solid residue was redissolved in toluene, added to the semi-preparative-scale Buckyprep HPLC column by injection (see below), and eluted with toluene at 5 mL min^–1^ (the HPLC trace is shown in Fig. S-20†). The fraction that eluted from 6.8 to 7.9 min was collected and evaporated to dryness under vacuum, yielding 3.9 mg of **2** (76% yield based on **1**).

### 3.3. Physicochemical methods

#### High-performance liquid chromatography

HPLC separation and analysis was carried out on samples exposed to air using a Shimadzu LC-6AD system with a SPD-20A UV/vis detector, a SPD-M20A diode array detector, and a CBM-20A communication bus module. The columns used were preparative- and semi-preparative-scale COSMOSIL Buckyprep columns (20 × 250 mm or 10 × 250 mm, respectively; Nacalai Tesque) and a COSMOSIL Buckyprep-M semi-preparative-scale column (10 × 250 mm, Nacalai Tesque) at a flow rate of 5 mL min^–1^ and observed at 370 nm unless otherwise indicated.

#### NMR and UV-vis spectroscopy and mass spectrometry

Fluorine-19 (376 MHz) and ^1^H (400 MHz) NMR spectra were recorded using a Varian INOVA 400 instrument using a 1 s relaxation time, 60° pulse angle, and 90/10 (v/v) PhCN/C_6_D_6_ or CDCl_3_ as the solvent with a trace amount of C_6_F_6_ (*δ*(^19^F) –164.90) added as the internal standard. Samples for spectra of **1** or **2** recorded at 23(1) °C were prepared without the exclusion of air; samples for spectra recorded at elevated temperatures and/or with added PS, CoCp_2_, or Sn_2_(*n*-Bu)_6_ were prepared anaerobically. The program MestReNova 8.1.1 was used to simulate the ^19^F NMR spectra of **1** and **2**. The uncertainties in the fitted *J*(FF) values are probably ±1 Hz. Mass spectra were recorded using a 2000 Finnigan LCQ-DUO mass-spectrometer with CH_3_CN used as the carrier solvent. UV-vis spectra of samples dissolved in toluene were recorded using a Cary 500 UV-vis-NIR spectrometer.

#### Electrochemistry

Cyclic and square-wave voltammograms were recorded in an inert-atmosphere glovebox using *ca.* 2 mM oDCB solutions containing 0.1 M N(*n*-Bu)_4_BF_4_ as the electrolyte, FeCp_2_ as the internal standard, and a PAR 263 potentiostat/galvanostat. The electrochemical cell was equipped with 0.125 mm diameter platinum working and counter electrodes and a 0.5 mm diameter silver wire quasi-reference electrode. The scan rate was 100 mV s^–1^.

#### Electron affinity measurement by low-temperature photoelectron spectroscopy (LT-PES)

The spectroscopy and procedures used were described previously.^4,41^ Anions **2** ^–^ were generated by electrospraying a 0.1 mM solution of **2** dissolved in toluene/acetonitrile to which a dilute acetonitrile solution of TDAE had been added dropwise until a color change from light brown to brown was observed. The anions were guided by quadrupole ion guides into a cryogenic ion trap, then transferred into the time-of-flight mass spectrometer. Mass-selected anions **2** ^–^ were intersected by a Nd:YAG laser (266 nm; 4.661 eV) in the photodetachment zone of the magnetic-bottle photoelectron analyzer. Photoelectrons were collected at nearly 100% efficiency, and the energy resolution (Δ*E*/*E*) obtained was *ca.* 2%. The gas-phase electron affinity (EA) of **2** was determined from the 0–0 transition in the 12 K LT-PES spectrum of the **2** ^–^ radical anion.

#### Time-resolved microwave conductivity (TRMC)

Samples for TRMC were 200–250 nm thick 1/1 (w/w) blended films of rr-P3HT and either PCBM or **2** prepared by spin coating 30 mg mL^–1^ oDCB solutions onto 1 × 2 cm quartz substrates in an inert-atmosphere glovebox. Neat P3HT films with similar thicknesses were prepared by spin coating 20 mg mL^–1^ oDCB solutions in the same way. The samples were placed in the resonance cavity at one end of a *ca.* 9 GHz X-band microwave waveguide. The films were exposed through the quartz substrate to 5 ns pulses of 500 nm photons using a Continuum Panther optical parametric oscillator pumped by the 355 nm harmonic of a Continuum Powerlite Q-switched Nd:YAG laser. The transient change in photoconductance (Δ*G*(*t*)) was measured by monitoring changes in the microwave power in the cavity (Δ*P*(*t*)) due to absorption of microwave photons by photogenerated electrons and holes in the thin film according to the equation:Δ*G*(*t*) = –(*K*(Δ*P*(*t*)/*P*)^–1^where *K* is a experimentally-determined calibration factor that depends on the microwave cavity resonance characteristics and the dielectric properties of the sample.^101^ The peak photoconductance, Δ*G*_peak_, is used to determine the yield of free carriers (*i.e.*, electron and holes), *φ*, times the sum of the free carrier mobilities, ∑*μ*, according to the equation:Δ*G*_peak_ = *βq*_e_ *I*_0_ *F*_A_ *φ*∑*μ*where *β* is the ratio of the dimensions of the cross-section of the waveguide (2.2 in our instrumentation), *q*_e_ is the charge on an electron, *I*_0_ is the incident photon flux, and *F*_A_ is the fraction of laser pump photons absorbed by the sample.

#### X-ray structure of 1,9-C_60_(*cyclo*-CF_2_(2-C_6_F_4_))

Crystals of **2** were grown by slow evaporation of a carbon disulfide solution. Data were collected on the Advanced Photon Source synchrotron instrument on beamline 15ID-B at Argonne National Laboratory, using a wavelength of 0.41328 Å, a diamond 111 monochromator, and a Bruker D8 goniometer. Unit cell parameters were obtained from a least-squares fit to the angular coordinates of all reflections. Intensities were integrated from a series of frames from *ω* and *φ* rotation scans. Absorption and other corrections were applied using TWINABS.^112^ The structure was solved as a non-merohedral twin using direct methods and refined on *F* ^2^ against one major and two minor twin components. Standard Bruker control and integration software (APEX II) was employed,^113,114^ and Bruker SHELXTL software was used with Olex 2 for the structure solution, refinement, and molecular graphics.^115,116^ For C_67_F_6_: *M* = 918.67, orthorhombic, *a* = 9.9998(6), *b* = 20.6538(12), *c* = 31.3512(18) Å, *V* = 6475.1(7) Å^3^, *T* = 15(2) K, space group *Pbca* (no. 61), *Z* = 8, 9670 reflections measured, 8340 unique which were used in all calculations. The final *R* and w*R* values are 0.073 (observed reflections) and 0.163 (all reflections), respectively.†

#### Computational methods

Optimization of molecular structures, transition states, and intrinsic reaction coordinate (IRC) calculations were performed *in vacuo* using the Priroda code^117,118^ at the OLYP level^119,120^ with the original TZ2P-quality basis set implemented in the code. Point energy calculations at the O3LYP/6-311G** level were performed using Firefly suite.^121^ We used the OLYP and O3LYP functionals because they have been shown to give good results for transition state energies for S_N_2 reactions.^122–124^ Solvation energies in benzonitrile (as a model for the experimental solvent mixture 90/10 (v/v) PhCN/C_6_D_6_) was computed using the C-PCM approach^125^ implemented in Firefly.
