# Supplementary material for: A faux hawk fullerene with PCBM-like properties
Source: Chem Sci. 2014 Dec 16;6(3):1801–15. doi: 10.1039/c4sc02970d (PMC5653957; doi:10.1039/c4sc02970d)
Supplement: Supplementary file 1 [file SC-006-C4SC02970D-s001.pdf]

Electronic Supplementary Material (ESI) for Chemical Science.
This journal is © The Royal Society of Chemistry 2015

Supporting Information for

A faux hawk fullerene with PCBM-like properties

Long K. San, Eric V. Bukovsky, Bryon W. Larson, James B. Whitaker, S. H. M. Deng, Nikos Kopidakis,* Garry Rumbles,* Alexey A. Popov,* Yu-Sheng Chen,* Xue-Bin Wang,* Olga V. Boltalina,* and Steven H. Strauss*

Abbreviations: **1** = 1,9-$C_{60}(CF_2C_6F_5)H$; **2** = 1,9-C_{60}(*cyclo*-$CF_2(2\text{-}C_6F_4)$)

Table of Contents

Figure S-1. Comparison of the structure of **2** with a faux hawk hairstyle S-2
Figure S-2. UV-vis spectra of purified **1** and **2** S-3
Table S-1. Distances and angles for the X-ray structure of **2** and OLYP DFT-optimized structures S-4
Figure S-3. Visual comparison of the X-ray and OLYP DFT-optimized structures of **2** S-5
Figure S-4. Enlarged version of the ^{19}F NMR spectrum of purified **1** S-6
Figure S-5. Enlarged version of the ^{19}F NMR spectrum of purified **2** S-7
Table S-2. ^{19}F NMR chemical shifts and coupling constants for **1** and **2** S-8
Figure S-6. Enlarged version of the S_NAr transformation **1** → **2** + HF (Figure 9 in the main text) S-9
Figure S-7. DFT distances and angles for the lowest-energy conformer of **1** S-10
Figure S-8. DFT distances and angles for a less-stable conformer of **1** S-11
Figure S-9. DFT distances and angles for the ground state [**1** − H]$^-$ anion S-12
Figure S-10. DFT distances and angles for the transition state [**1** − H]$^-$ anion S-13
Figure S-11. DFT distances and angles for the intermediate state [**1** − H]$^-$ anion S-14
Figure S-12. DFT distances and angles for **2** S-15
Table S-3. DFT relative energies for other functionals and O3LYP//OLYP solvation energies S-16
Figure S-13. Molecular structures of **2** and PCBM S-17
Solid-state packing, molecular and X-ray structure comparisons of **2** and PCBM S-18
Figure S-14. The centroid packings in the single-crystal X-ray and PXRD structures of PCBM S-21
Figure S-15. Solid-state packing and centroid···centroid distances for **2** S-22
Figure S-16. Solid-state packing and centroid···centroid distances for PCBM S-23
Figure S-17. Unit cell and packing pattern of the C_{60} cage centroids in the structure of **2** S-24
Figure S-18. C_{60} cage centroid packing patterns for PCBM, and related X-ray structures S-25
Table S-4. Fullerene cage centroid···centroid distances for **2**, PCBM, and related X-ray structures S-26
Figure S-19. Drawings of the DFT-optimized structures of PCBM, iso-PCBM, and **2** S-27
Figure S-20. HPLC trace of the "**1** plus excess Proton Sponge in PhCN" reaction mixture S-28
References for Supplementary Information S-29

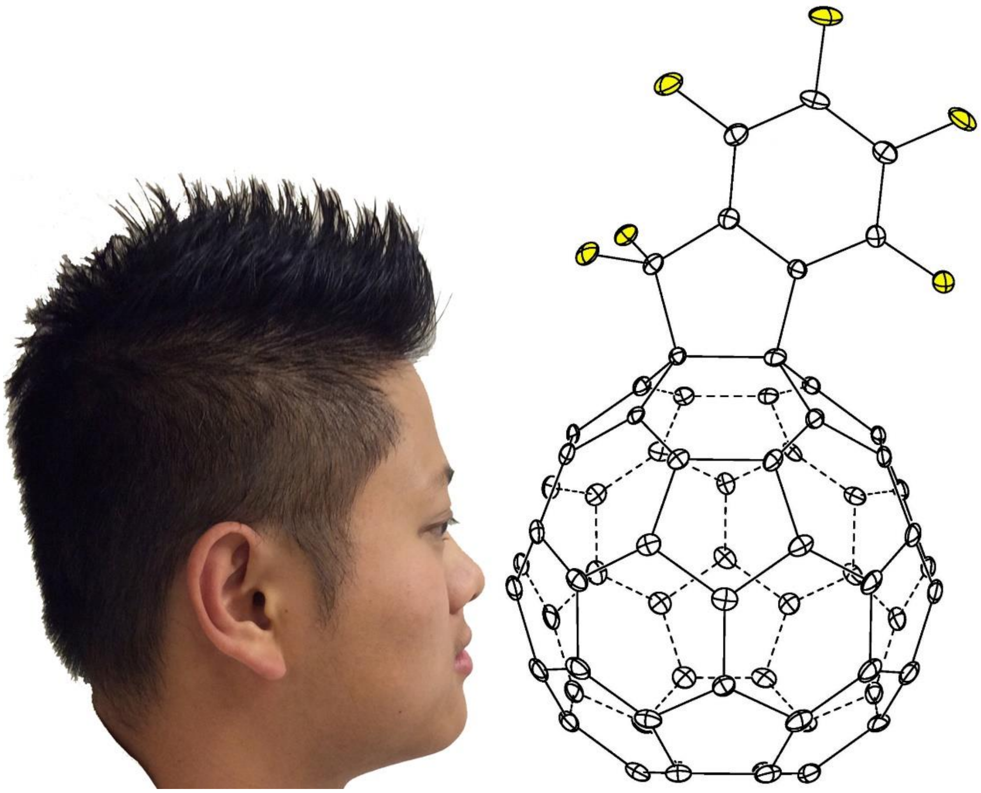

Figure S-1. Comparison of the structure of **2** (50% probability ellipsoids) with a photograph of a faux hawk hairstyle. The resemblance led to the "faux hawk" nickname for **2**.

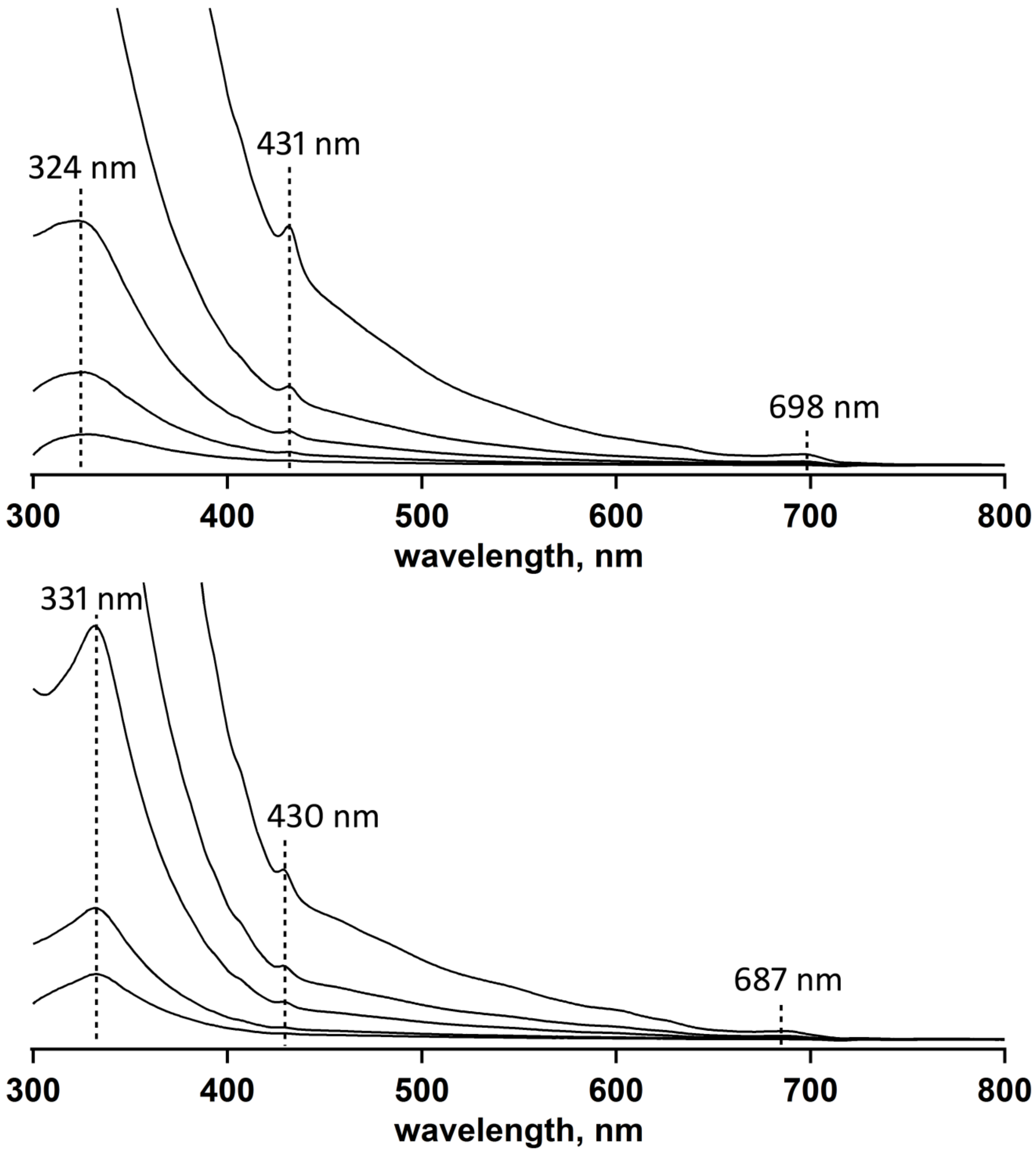

Figure S-2. UV-vis spectra of **1** (top) and **2** (bottom) dissolved in toluene. Successive dilutions allow key absorption features to be seen.

Table S-1. Interatomic distances (Å) and angles (deg) for the X-ray structure of **2** and the OLYP DFT-optimized structures of 1,9- and 1,2-C_{60}(*cyclo*-CF_2(2-C_6F_4))[a]

parameter	X-ray **2**	DFT 1,9-C_{60}(*cyclo*-CF_2(2-C_6F_4))	DFT 1,2-C_{60}(*cyclo*-CF_2(2-C_6F_4))
C1–C9	1.610(5)	1.608	1.636
C1–C2	1.538(6)	1.531	1.531
C2–C7	1.374(6)	1.395	1.395
C7–C8	1.482(5)	1.496	1.503
C8–C9	1.572(5)	1.587	1.589
F1···F6	2.997(6)	3.088	3.049
F2···F6	3.151(6)	3.088	3.141
C2–C1–C9	102.6(3)	103.6	103.2
C2–C1–C	109.5(3); 115.8(3)	114.3 × 2	112.7; 114.9
C8–C9–C1	105.2(3)	105.5	105.1
C8–C9–C	109.2(3); 113.6(3)	114.5 × 2	112.0; 113.4
C1–C2–C7	113.0(3)	112.9	113.2
C2–C7–C6	121.4(4)	121.8	121.5
C2–C7–C8	111.7(4)	111.6	111.9
C7–C8–C9	106.4(3)	106.4	106.3

[a] The X-ray structure of **2** and the DFT 1,9- isomer which has the faux hawk substituent attached to a C_{60} cage bond shared by two hexagons. The hypothetical DFT 1,2- isomer (not observed) has the substituent attached to a C_{60} cage bond shared by a hexagon and a pentagon.

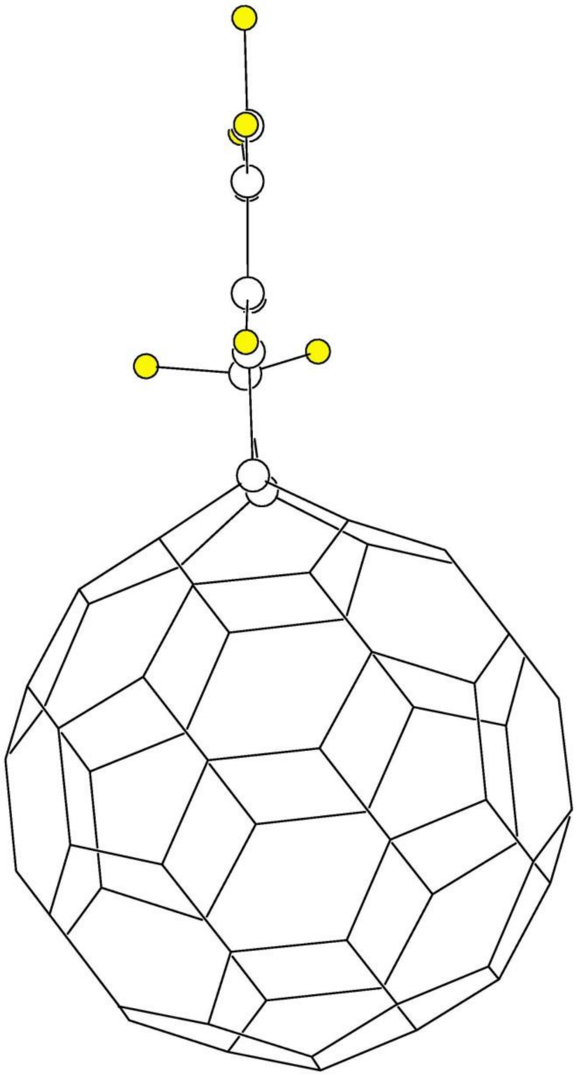

Figure S-3. Visual comparison of the X-ray structure (left) and OLYP DFT-optimized structure (right) of **2** with respect to the planarity of the faux hawk substituent and its placement perpendicular to the surface of the C_{60} cage. The faux hawk C and F atoms are shown as spheres of arbitrary size; the F atoms are highlighted in yellow.

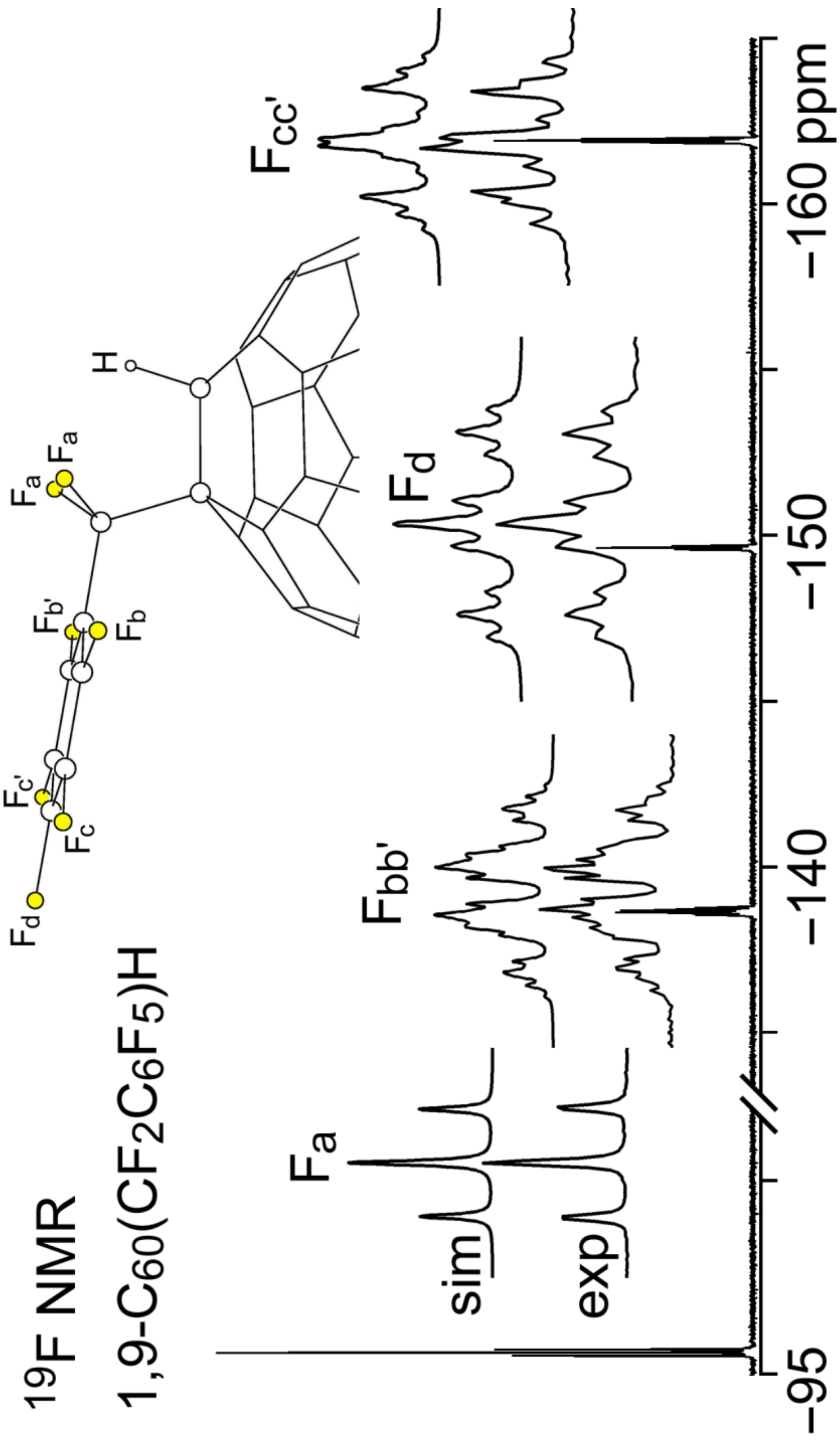

Figure S-4. Experimental and simulated 376 MHz ^{19}F NMR spectra of HPLC-purified **1** in $CDCl_3$ (C_6F_6 int. std. (δ −164.9)). This is Figure 3 in the main text.

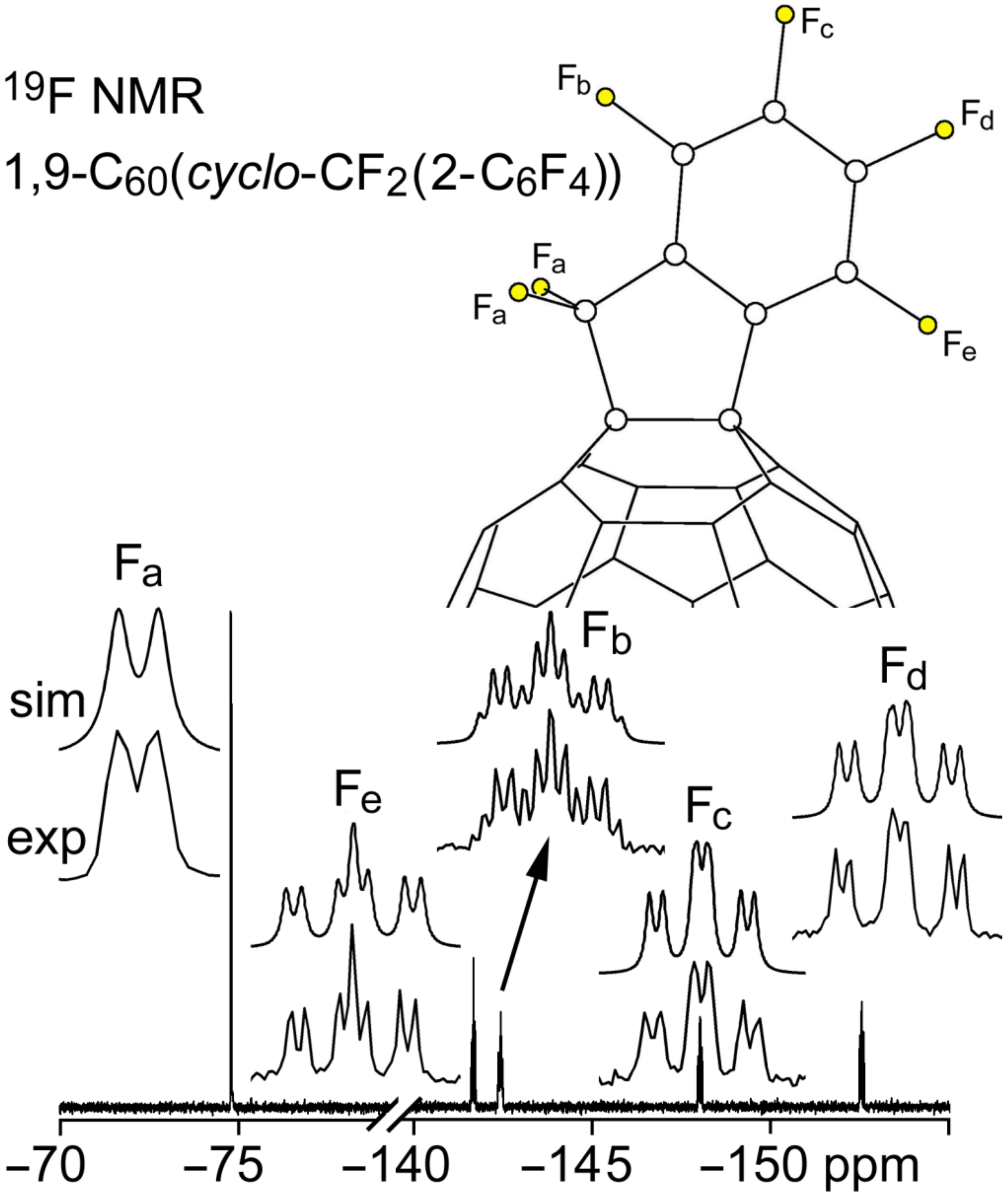

Figure S-5. Experimental and simulated 376 MHz ^{19}F NMR spectra of HPLC-purified **2** in $CDCl_3$ (C_6F_6 int. std. (δ −164.9)). This is Figure 4 in the main text.

Table S-2. 376 MHz ^{19}F NMR δ and *J*(FF) values for **1** and **2** (δ(C_6F_6) = −164.9)

	1,9-$C_{60}(CF_2C_6F_5)H$ (**1**)	1,9-C_{60}(*cyclo*-CF_2(2-C_6F_4)) (**2**)
δ(F_a)	−95.5 (t)	−74.8 (d)
$J(F_aF_{bb'})/J(F_aF_b)$	30	5.5
δ($F_{bb'}$)/δ(F_b)	−138.7 (qt)	−142.4 (m)
$J(F_bF_{b'})$	5	
$J(F_{bb'}F_d)/J(F_bF_d)$	5.5	6
$J(F_bF_c)$	26	18
$J(F_bF_{c'})$	7	
$J(F_bF_e)$		23
δ($F_{cc'}$)/δ(F_c)	−161.9 (m)	−148.0 (td)
$J(F_{cc'}F_d)/J(F_cF_d)$	22	20
$J(F_cF_e)$		5
δ(F_d)	−149.6 (tt)	−152.6 (td)
$J(F_dF_e)$		18
δ(F_e)		−141.7 (tt)

[a] Coupling constants (Hz) are estimated (±1 Hz) from simulated spectra. [b] d = doublet; t = triplet; q = quartet; m = multiplet. The solvent was $CDCl_3$.

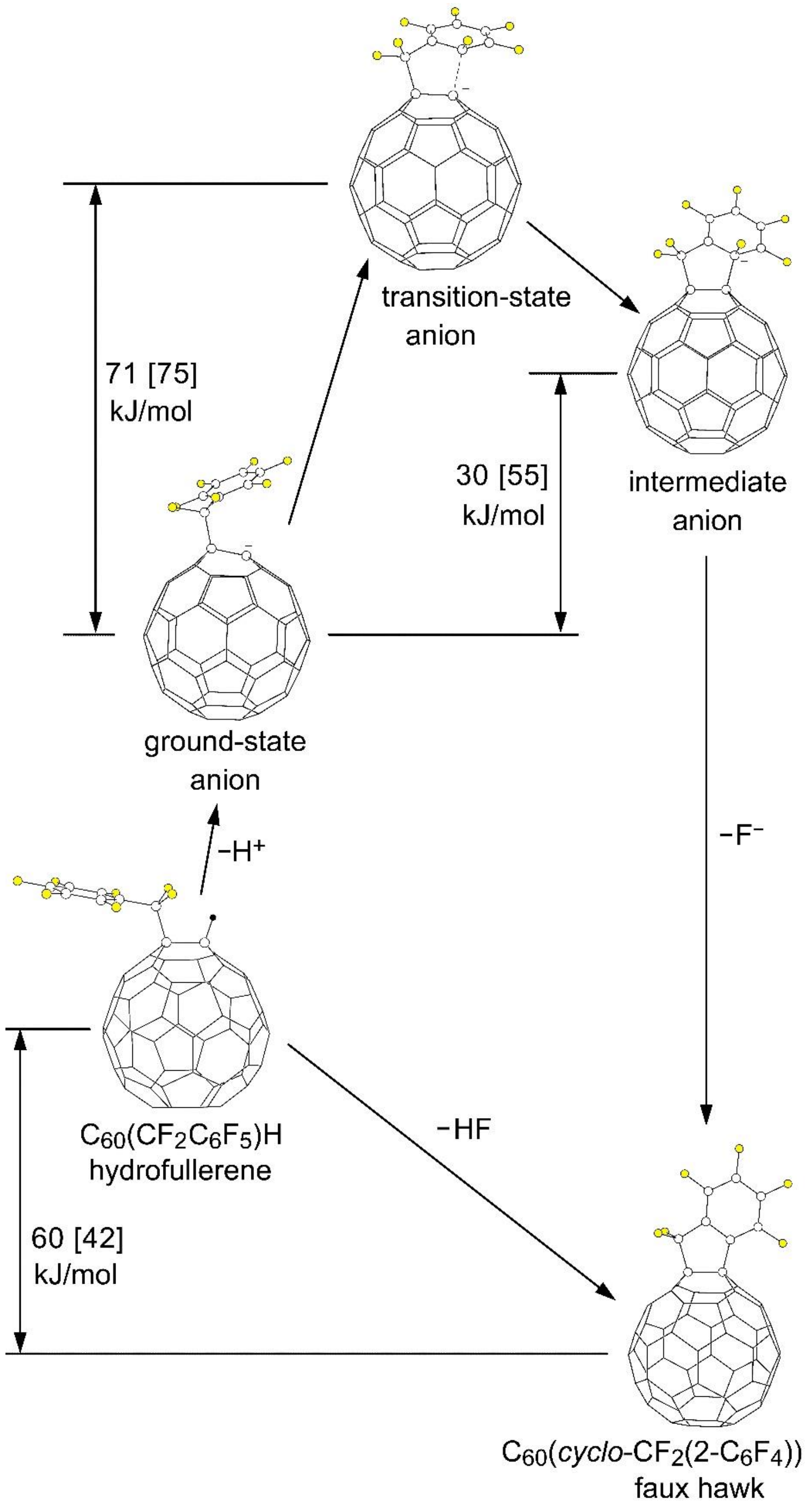

Figure S-6. Proposed S_NAr transformation of **1** into **2** + HF. The O3LYP/OLYP DFT-predicted energy changes shown, which are not to scale on the vertical axis, are for (i) a dielectric continuum equivalent to benzonitrile (no brackets) and (ii) the gas phase (square brackets). This is Figure 9 in the main text.

OLYP DFT-optimized gas-phase structure of 1,9-$C_{60}(CF_2C_6F_5)H$ (**1**)

This hydrofullerene derivative is the precursor to the faux hawk fullerene.

This drawing represents the lowest energy conformation

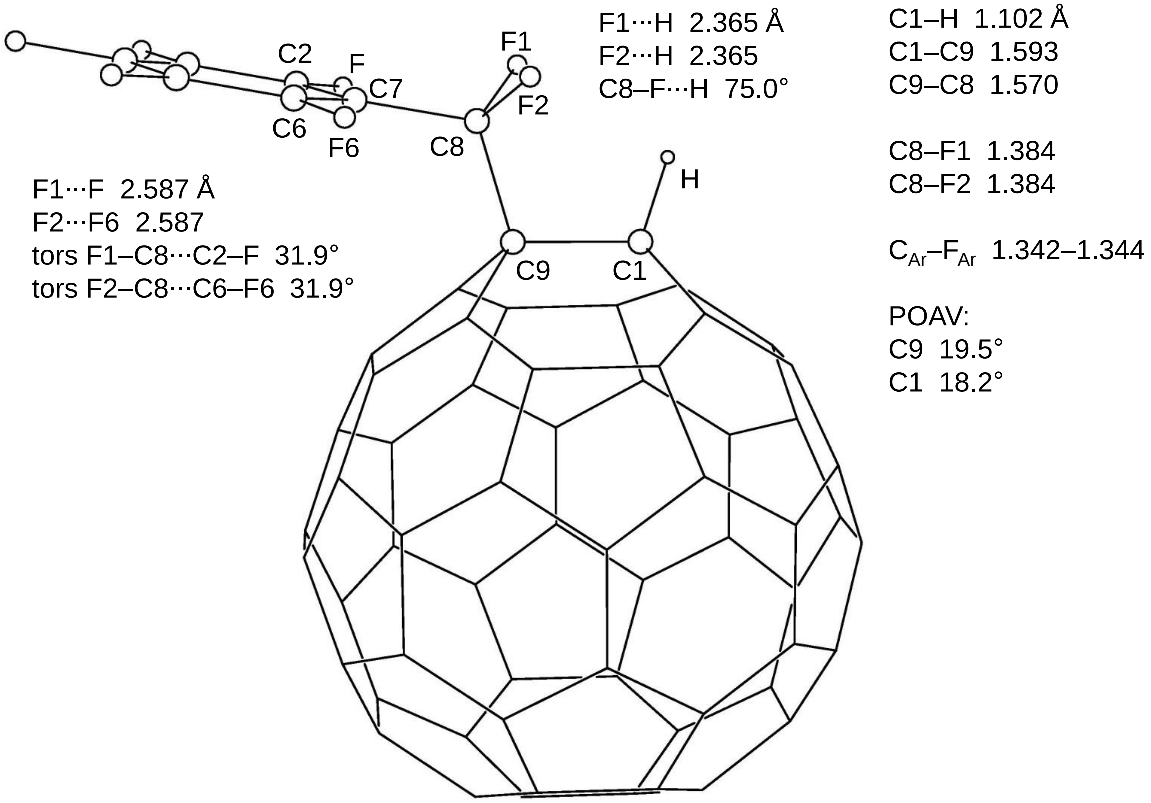

Figure S-7. Distances and angles for the gas-phase OLYP DFT-optimized lowest-energy conformer of **1**.

Alternate OLYP DFT-optimized gas-phase structure of 1,9-$C_{60}(CF_2C_6F_5)H$ (**1**)

This conformer is a false minimum and was found to be 6.1 kJ/mol less stable than the conformer the one on the previous page

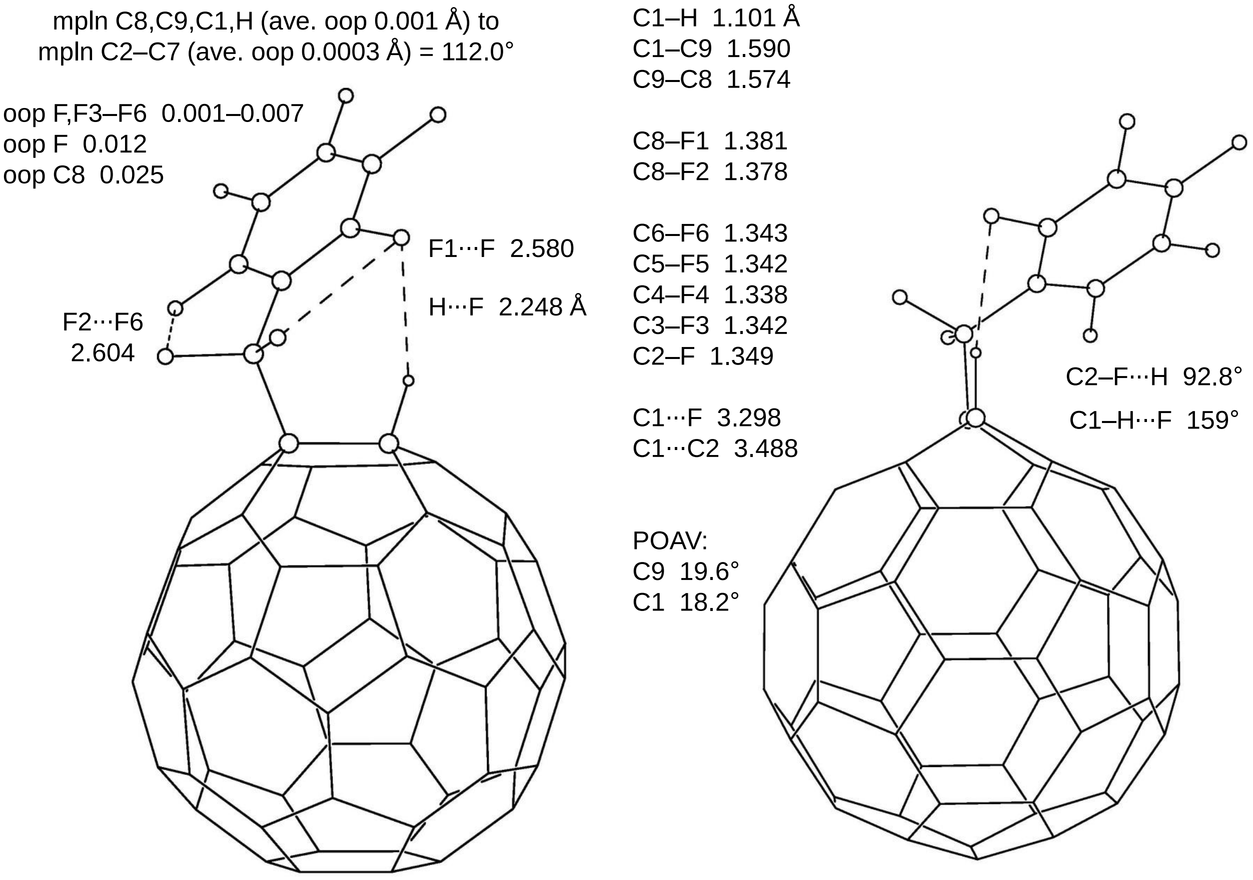

Figure S-8. Distances and angles for a gas-phase OLYP DFT-optimized conformer of **1** that is 6.1 kJ/mol less stable than the structure shown in Figure S-7 on the previous page.

OLYP DFT-optimized gas-phase structure of the ground state [**1** − H]$^{-}$ anion

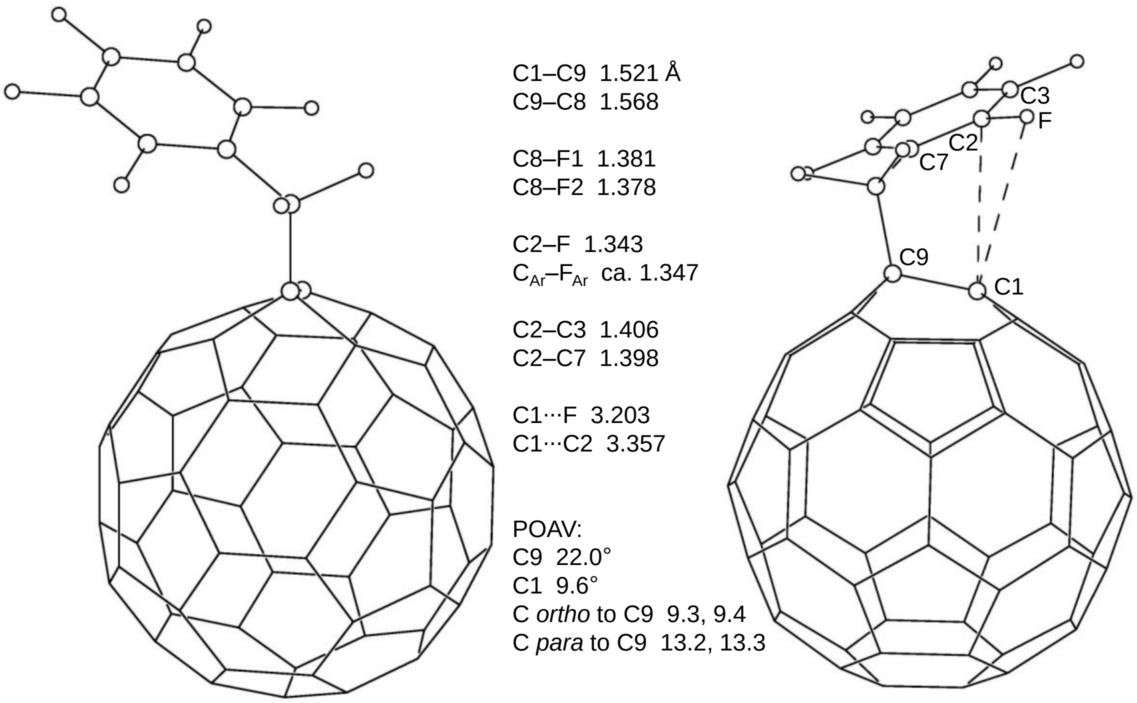

Figure S-9. Distances and angles for the gas-phase OLYP DFT-optimized structure of the ground state [**1**−H]$^{-}$ anion.

OLYP DFT-optimized gas-phase structure of the transition state [**1** − H]⁻ anion

(aka the pre-faux hawk anionic transition state)

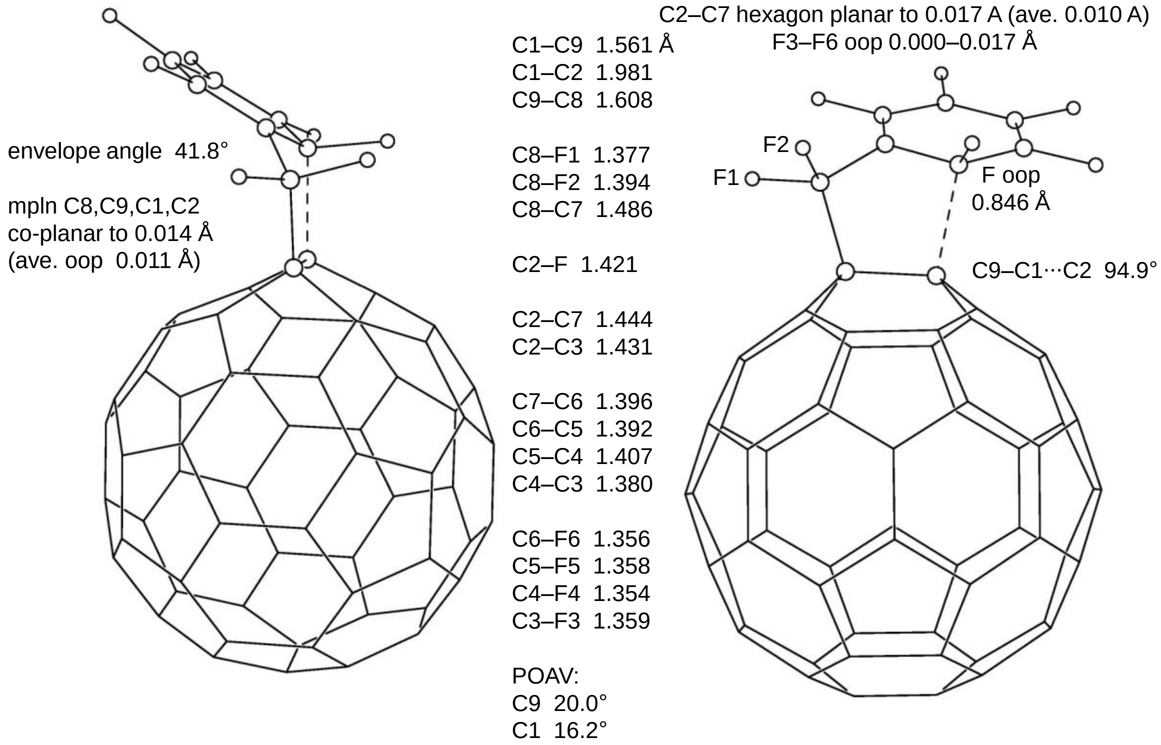

Figure S-10. Distances and angles for the gas-phase OLYP DFT-optimized structure of the transition state [**1**−H]$^{-}$ anion.

OLYP DFT-optimized gas-phase structure of the intermediate [**1** − H]⁻ anion
(the proposed Meisenheimer intermediate anion)

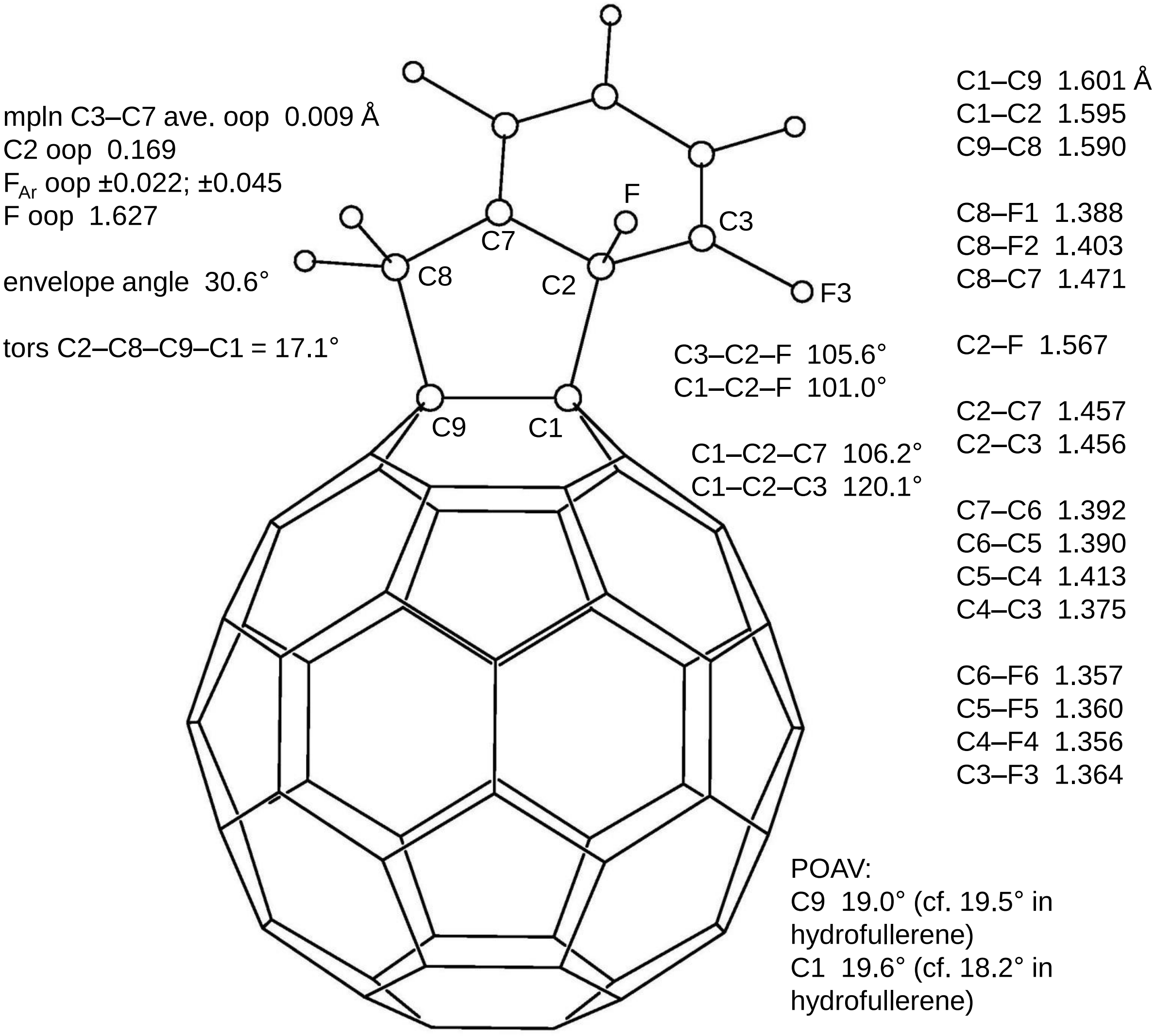

Figure S-11. Distances and angles for the gas-phase OLYP DFT-optimized structure of the intermediate state [**1**−H]⁻ anion.

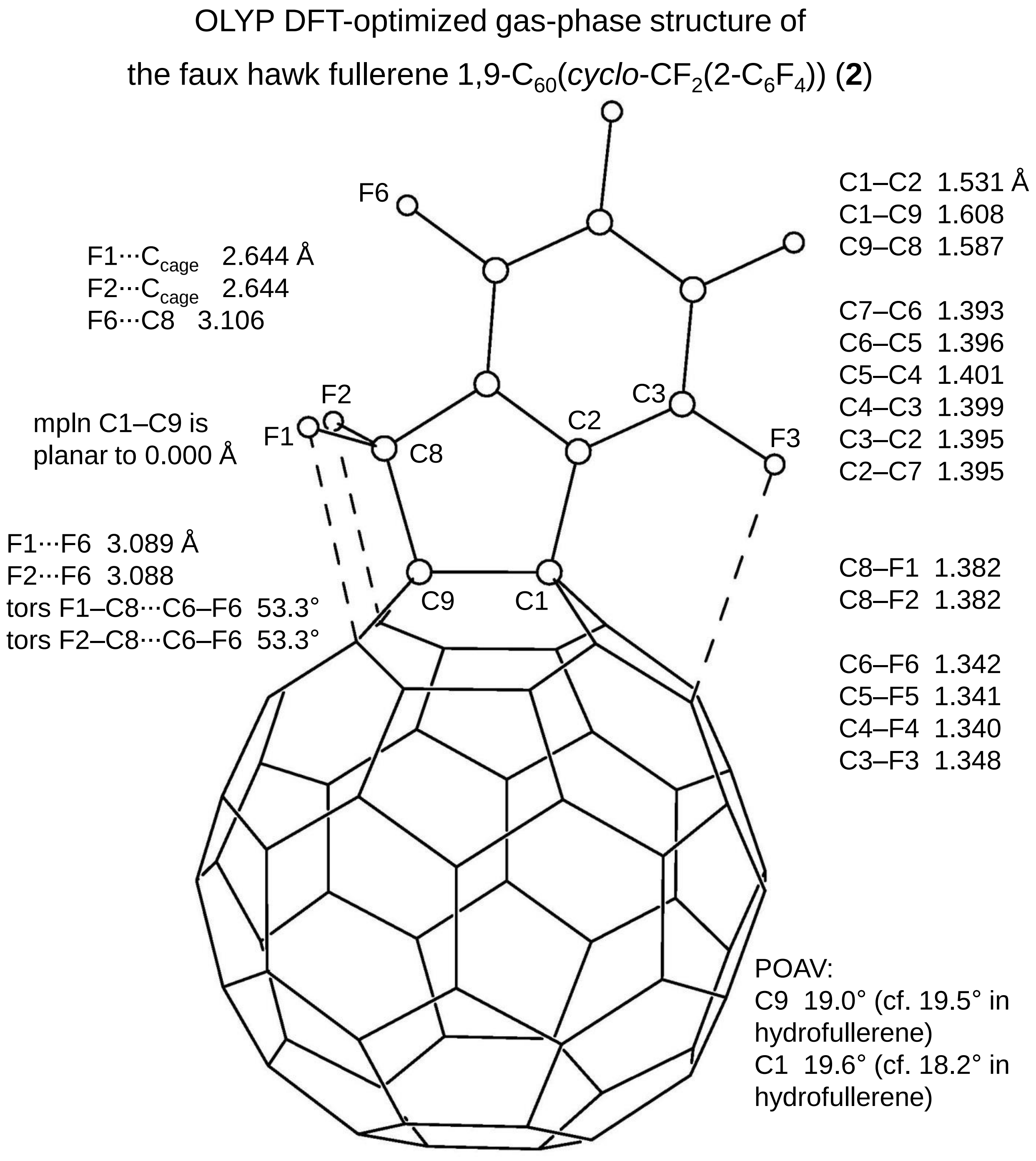

Figure S-12. Distances and angles for the gas-phase OLYP DFT-optimized structure of **2**.

Table S-3. DFT relative energies (kJ/mol) for other functionals and O3LYP//OLYP solvation energies[a]

functional	ground state **1**$^-$ anion	transition state **1**$^-$ anion	intermediate **1**$^-$ anion
PBE	0.0	72.4	49.8
OLYP	0.0	85.3	67.3
O3LYP//OLYP solvation energy	123.2	128.1	148.7

[a] The designations "ground state," "transition state," and "intermediate" are the same as those used in Figure S-6 (Figure 10 in the main text). The O3LYP//OLYP relative energies, in the gas-phase and in a PhCN-like dielectric continuum, are shown in Figure S-6.

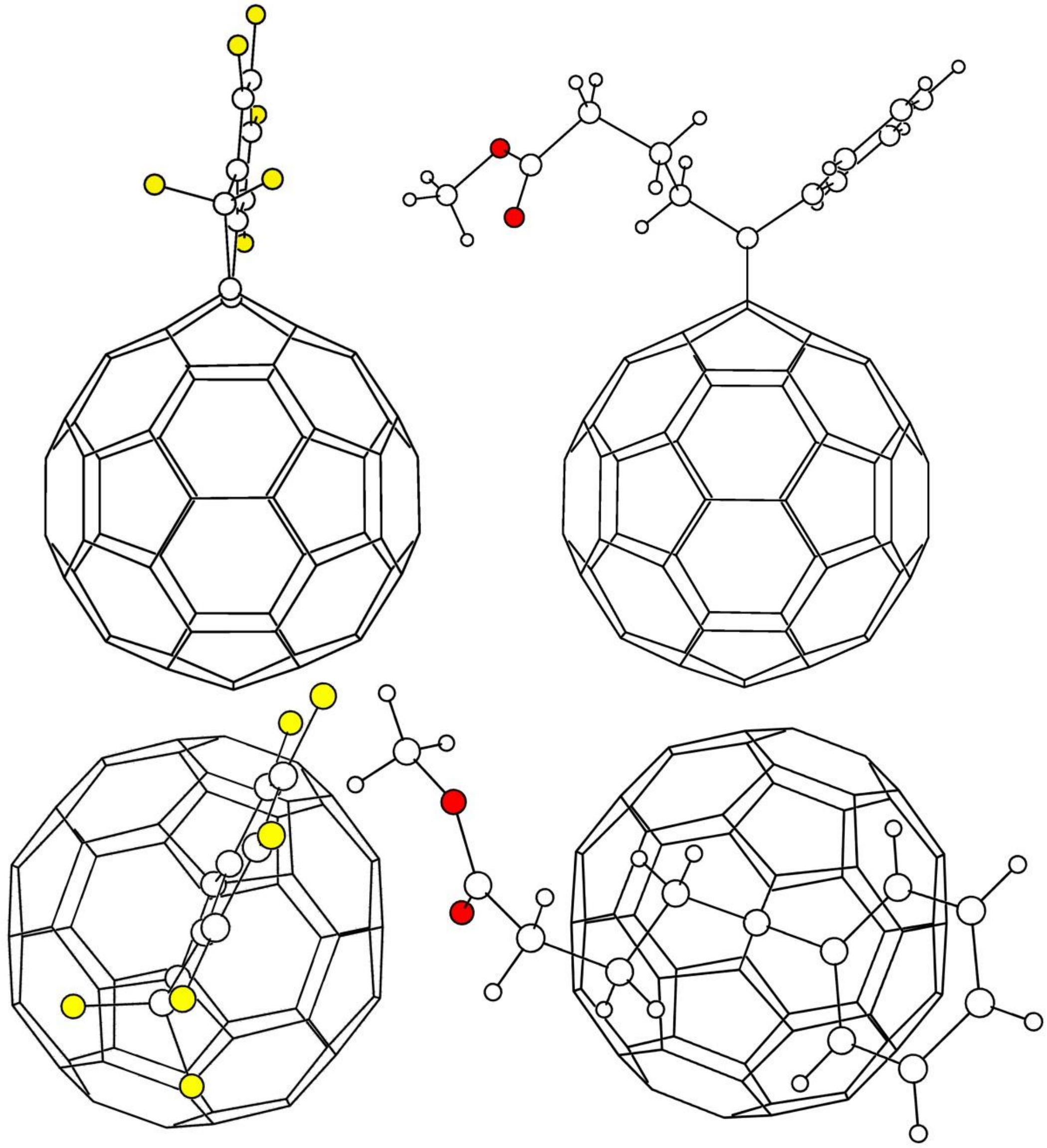

Figure S-13. Comparison of the molecular structures of faux hawk fullerene **2** (left, this work) and PCBM (right, ref 1). The large and small white spheres represent C and H atoms, respectively; the red and yellow spheres represent O and F atoms, respectively. The fullerene cages and the substituent atoms in the two structures are scaled equally.

Solid-state packing, molecular and X-ray structure comparisons of 2 and PCBM

There are two solvent-free X-ray structures of PCBM: a single-crystal structure determined using data collected at 100(2) K[1] and a structure determined by powder X-ray diffraction data collected at 298(2) K.[2] Both have the same space group, the same nearest-neighbor crystal packing (see Figure S-13), and, despite the difference in temperature, have densities (i.e., unit cell volumes) that differ by only 0.64(3)%. This is somewhat surprising because most organic crystals exhibit a larger percentage-increase in density between 300 and 100 K, typically 3–6%.[3] For example, the density increases over this temperature range for benzene, naphthalene, and the *F*3*m*3 polymorph of C_{60} are 5.1, 7.8, and 2.0%, respectively.[3] This may indicate that solvent-free PCBM is packed as tightly as possible at 25 °C, even more so than C_{60}.

The molecular structures of **2** and the 100 K single-crystal structure PCBM[1] are shown side-by-side in Figure S-14. The two substituents have nearly the same number of non-hydrogen atoms, 13 for **2** and 14 for PCBM, but the faux hawk substituent is clearly the more compact. The 1.632(2) Å C1–C9 bond in PCBM is only marginally longer than the 1.610(5) Å distance in **2**, and fullerene cage atoms C1 and C9 are only slightly less pyramidalized in PCBM (POAV θ_p = 17.1° × 2) than in **2** (θ_p = 18.9 and 19.1°).

The solvent-free solid-state packing of **2** and PCBM[1] are shown in Figures S-15 and S-16, respectively. In both cases the C_{60} cage centroids (⊙) form rigorously-planar layers that are stacked in the third dimension. (In the structure of **2**, the stacking direction is parallel to the crystallographic *c* axis, as shown in Figure S-17.) Significantly, the numbers of nearest neighbor molecules in the two structures are different. There are only seven (7) nearest neighbor fullerene molecules in crystalline solvent-free PCBM, with ⊙···⊙ distances of 9.95–10.28 Å. The mean and median distances are 10.17 and 10.24 Å, respectively. On the other hand, there are ten (10) nearest neighbors in the structure of **2**, with ⊙···⊙ distances of 9.74–10.34 Å. The mean and median distances are 10.09 and 10.05 Å, respectively. The result is that the density of crystalline **2**, 1.885 g cm^{-3}, is 15.6% higher than the 1.631 g cm^{-3} density of solvent-free PCBM, even though the molar masses of the two compounds, 918.67 g mol^{-1} for **2** and 910.83 g mol^{-1} for PCBM, differ by only 1.1%. Although the diffraction data reported here for **2** were collected at 15(2) K, unit cell parameters were also determined at 120(2) K, and the unit cell volume was only 0.64% larger than at 15(2) K. If the 120 K density is considered, then the density of crystalline **2** is 14.8% higher than crystalline solvent-free PCBM at 100 K.

It is widely believed that the aggregation behavior of OPV acceptor fullerenes in the solid state, especially the number of electronically coupled nearest neighbors and their three-dimensional arrangement, are among the key factors that determine charge transport properties in the fullerene domains in Type II heterojunction solar cells.[1,2,4-13] Accordingly, the determination of the number of nearest neighbor fullerenes is important, and it depends on the choice of the maximum relevant ⊙···⊙ distance beyond which fullerene–fullerene electronic coupling is probably negligible. After the seven closest PCBM molecules surrounding each molecule of PCBM in the solvent-free structure,[1] shown in

Figure S-16, the two next shortest ⊙···⊙ distances are 11.61 and 13.23 Å. On what basis did we decide whether or not the 11.61 Å molecule should be considered to be an electronically-relevant nearest neighbor? The criterion we propose is as follows. We consider the 11.61 Å distance to be too long for effective electronic coupling because the closest $C_{cage}\cdots C_{cage}$ distance between these two PCBM molecules is 5.03 Å (and these two C_{cage} atoms are close to lying on the ⊙···⊙ vector). In contrast, the closest $C_{cage}\cdots C_{cage}$ distances for PCBM molecules with ⊙···⊙ separations of 10.28 and 10.24 Å are 3.23 and 3.32 Å, respectively, approximately the same as the 3.35 Å interplanar separation in graphite.[14] For **2**, the closest C···C distance between two molecules with separations of 10.34 Å is 3.38 Å, and the next closest ⊙···⊙ distances are 14.00 Å. Therefore, the two centroids that are 10.34 Å from the central centroid in Figure S-15 belong to faux hawk molecules counted among the ten nearest neighbors around each faux hawk molecule.

Interestingly, the perpendicular spacings between the rigorously-planar layers of centroids are smaller, not larger, in the structure of solvent-free PCBM, 5.89 and 6.46 Å, than in the structure of **2**, 6.96 and 8.71 Å. Due to offsets of the C_{60} centroid layers relative to one another, the interlayer spacings are not an important metric from the standpoint of electron mobility. The ⊙···⊙ distances and their three-dimensional arrangement are important.

It may come as a surprise to many readers that the ⊙···⊙ distances in PCBM crystals containing solvent molecules can be, on average, shorter, not longer, than in the solvent-free structure discussed above, *even when there are as many PCBM nearest neighbors*. In the 123 K single-crystal structure of PCBM·$0.5CS_2$,[6] seven ⊙···⊙ distances span the range 9.86–10.27 Å and average 10.08 Å. There are two unique PCBM molecules in the 90 K structure of PCBM·$0.5C_6H_5Cl$, they both have seven nearest neighbors, and the mean ⊙···⊙ distances are 10.01 and 10.02 Å (the two ranges are 9.84–10.14 and 9.95–10.06 Å, respectively).[10] In the 123 K structure of ThCBM·$1.25CS_2$, in which a nearly-isosteric thienyl five-membered ring has replaced the phenyl group in PCBM, there are two unique ThCBM molecules.[6] One has seven nearest neighbors with a mean ⊙···⊙ distance of 10.03 Å (the range is 9.98–10.19 Å) and the other has *ten* nearest neighbors with a mean ⊙···⊙ distance of 9.99 Å (the range is 9.84–10.19 Å).[6] Finally, in the 90 K structure of PCBM·*o*-$C_6H_4Cl_2$, the one exception that proves the rule, there are only six, not seven, ⊙···⊙ distances, although the mean distance is still small, only 10.01 (the range is 10.00–10.22 Å).[10] All of the individual ⊙···⊙ distances as well as the mean distances for the structures just discussed are listed in Table S-4. Whatever space is taken up by solvent molecules in structures of PCBM and related molecules, the fullerene–fullerene interactions can be as strong and as extensive as in solvent-free structures. It remains to be seen whether the presence of *all* types of solvent molecules, not just the ones examined so far, and/or the presence of other so-called "impurities" in fullerene domains *always* have a deleterious effect on electron mobility in blended donor-acceptor thin films and/or on power conversion efficiencies of OPV devices made with such films.

As stated above, and as previously suggested by others,[1,2,4-10] whether or not the nearest-neighbor ⊙···⊙ vectors in a fullerene domain point in three dimensions, and not just in two-dimensional layers, should affect electron mobility in a three-dimensional domain as much as the number of and the distances to the nearest neighbor fullerene molecules. The three-dimensional nature of the packing patterns in the X-ray structures of **2** and solvent-free PCBM are shown in Figures S-15 and S-16. The corresponding figures for the X-ray structures of PCBM·$0.5CS_2$, PCBM·$0.5C_6H_5Cl$, PCBM·*o*-$C_6H_4Cl_2$, and ThCBM·$1.25CS_2$ are shown in Figure S-18. In all cases but one, the packing is three dimensional. In the case of PCBM·*o*-$C_6H_4Cl_2$, the packing is essentially two dimensional and, as pointed out by the authors of the paper reporting the structure, this should hamper three-dimensional-hopping electron transport.[99]

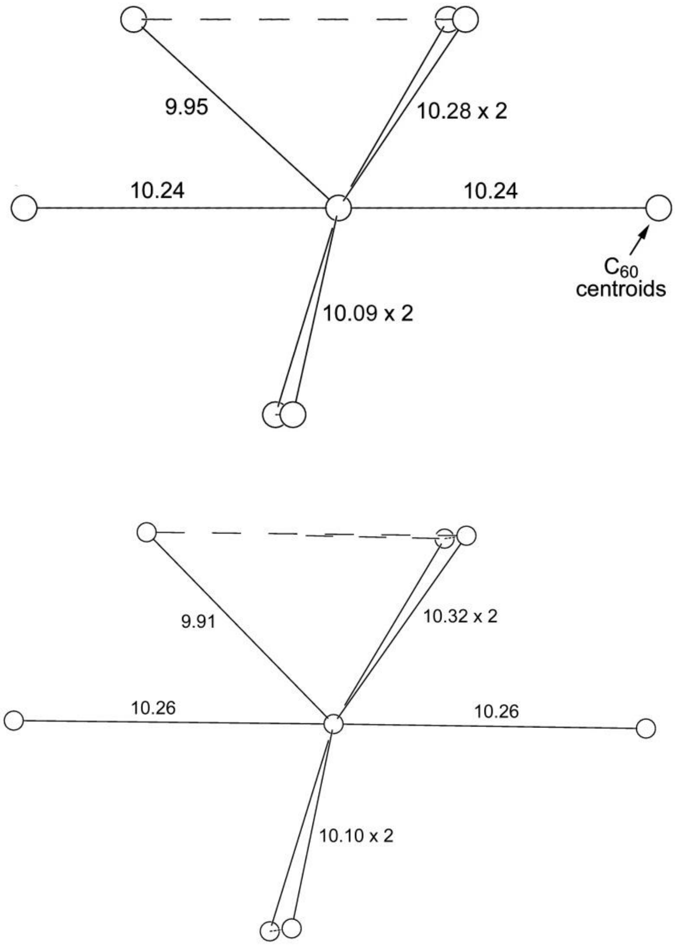

Figure S-14. Comparison of the nearest-neighbor centroid packing patterns in the single-crystal X-ray structure (top;釘ernò, G.; Warren, A. J.; Spencer, J.; Evans, G.; García Sakai, V.; Blumberger, J.; Cacialli, F. *J. Mater. Chem.* **2013**, *1*, 5619; data collected at 100 K) and the powder X-ray diffraction structure (bottom; Casalegno, M.; Zanardi, S.; Frigerio, F.; Po, R.; Carbonera, C.; Marra, G.; Nicolini, T.; Raos, G.; Meille, S. V. *Chem. Commun.* **2013**, *49*, 4525; data collected at 300 K) of solvent-free PCBM (PCBM = phenyl-C_{61}-butyric acid methyl ester). The averages of the seven nearest-neighbor distances are 10.17 (top) and 10.18 Å (bottom).

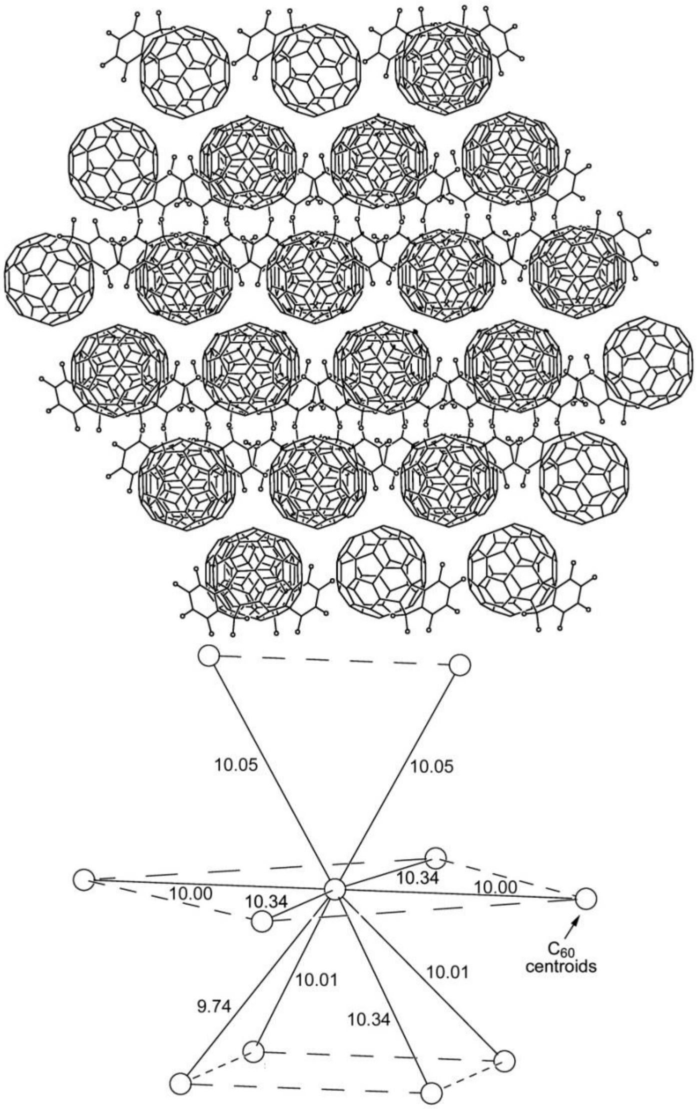

Figure S-15. The packing of molecules of the faux hawk fullerene (**2**) determined by single-crystal X-ray diffraction. The molecules are arranged in layers with rigorously co-planar C_{60} cage centroids. The layers are stacked in the direction parallel to the long axis of the page, which in this case is parallel to the crystallographic *c* axis. Each faux hawk fullerene molecule is surrounded by 10 nearest neighbor molecules with C_{60} centroid···centroid distances that range from 9.74 to 10.34 Å (ave. 10.09 Å). The lower centroid diagram is only slightly turned and tilted from the orientation of molecules in the upper packing diagram for clarity.

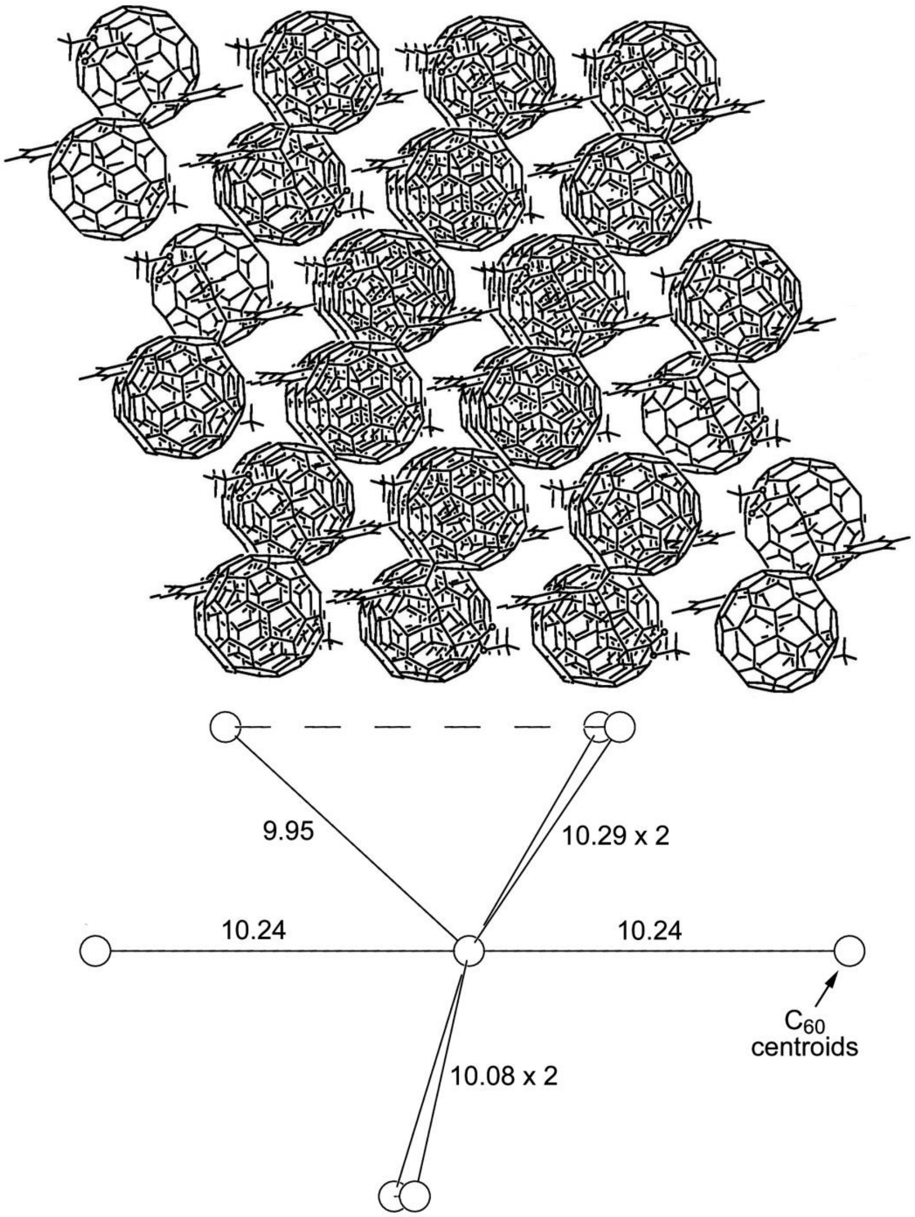

Figure S-16. The packing of molecules of PCBM in the solvent-free structure determined by single-crystal X-ray diffraction (ref 1). The molecules are arranged in layers with rigorously co-planar C_{60} cage centroids. The layers are stacked in the direction parallel to the long axis of the page. Each PCBM molecule is surrounded by 7 nearest neighbor molecules with centroid···centroid distances that range from 9.95 to 10.28 Å (ave. 10.17 Å). The orientations of the centroids in the lower diagram and the molecules in the upper packing diagram are the same.

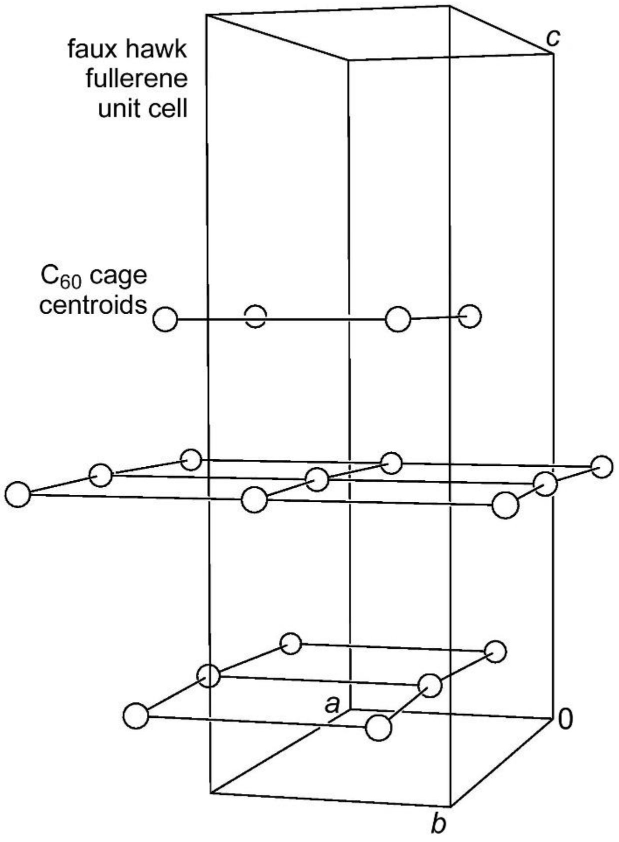

Figure S-17. Unit cell and packing pattern of the C_{60} cage centroids in the crystal structure of faux hawk fullerene **2**. The rigorously planar, approximately square arrays of centroids are stacked along the crystallographic *c* axis.

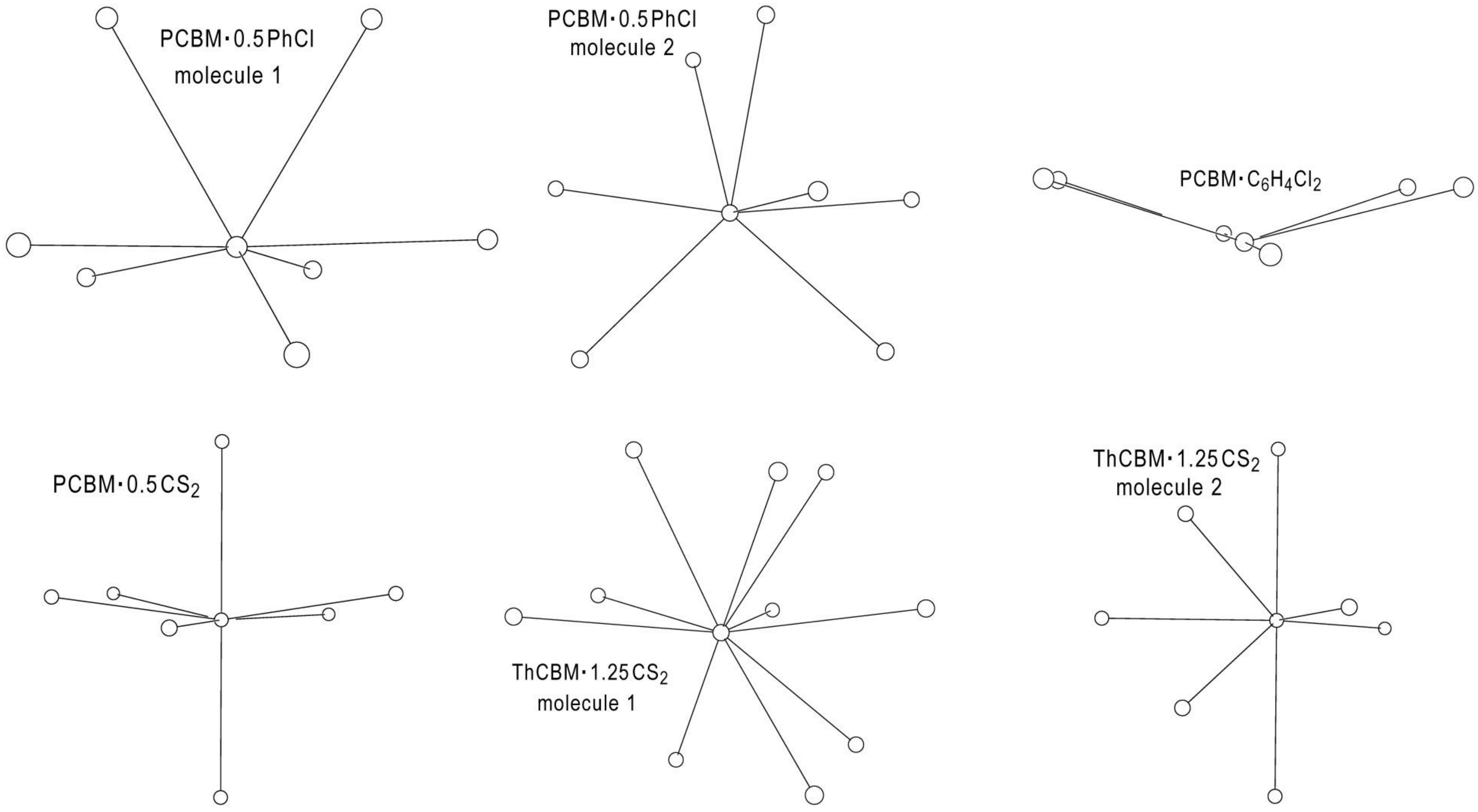

Figure S-18. Packing patterns of the C_{60} cage centroids in the X-ray structures of PCBM·0.5C_6H_5Cl (two unique molecules in the asymmetric unit), PCBM·*o*-$C_6H_4Cl_2$, PCBM·0.5CS_2, and ThCBM·1.25CS_2 (two unique molecules in the asymmetric unit). In all cases except for PCBM·*o*-$C_6H_4Cl_2$, the packing of the centroids and hence the packing of the fullerene molecules is three dimensional. The literature references for these structures are given in the main text.

Table S-4. Centroid···centroid distances in solvent-free and solvent-included fullerene X-ray structures[a,b]

9.739	9.952	9.910	9.843	9.945	10.000	9.840	9.982
10.000	10.092	10.096	9.945	9.948	10.000	9.903	9.982
10.000	10.092	10.096	9.994	9.948	10.104	9.930	9.998
10.010	10.278	10.262	10.046	9.994	10.183	9.982	10.018
10.010	10.278	10.262	10.054	10.046	10.183	9.982	10.021
10.046	10.236	10.322	10.101	10.064	10.217	9.997	10.026
10.046	10.236	10.322	10.138	10.101		9.998	10.189
10.331						10.018	
10.342						10.026	
10.342						10.189	
10.087	10.166	10.181	10.017	10.007	10.115	9.987	10.031
faux hawk	Paterno	Casalegno	Rispens et al.	Rispens et al.	Rispens et al.	Choi et al.	Choi et al.
(this work)	et al.	et al.	PCBM.0.5PhCl	PCBM.0.5PhCl	PCBM.*o*-$C_6H_4Cl_2$	ThCBM.1.25CS_2	ThCBM.1.25CS_2
	single-	PXRD	molecule 1	molecule 2			
	crystal	solvent-					
	solvent-	free PCBM					
	free PCBM						

[a] The mean values are given in the line just above the text. [b] The literature references are given below.

Paternò et al. = Paternò, G.; Warren, A. J.; Spencer, J.; Evans, G.; García Sakai, V.; Blumberger, J.; Cacialli, F. *J. Mater. Chem.* **2013**, *1*, 5619.

Casalegno et al. = Casalegno, M.; Zanardi, S.; Frigerio, F.; Po, R.; Carbonera, C.; Marra, G.; Nicolini, T.; Raos, G.; Meille, S. V. *Chem. Commun.* **2013**, *49*, 4525.

Rispens et al. = Rispens; M. T.; Meetsma, A.; Rittberger, R.; Brabec, C. J.; Sariciftci, N. S.; Hummelen, J. C. *Chem. Commun.* **2003**, 2116.

Choi et al. = Choi, J. H.; Honda, R.; Seki, S.; Fukuzumi, S. *Chem. Commun.* **2011**, *47*, 11213.

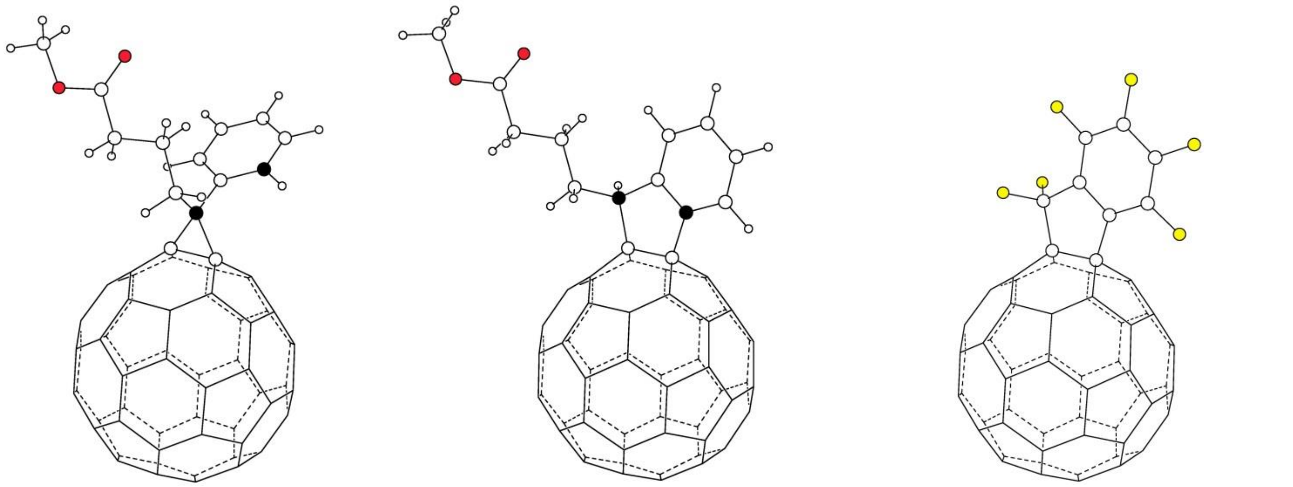

PCBM iso-PCBM faux hawk (**2**)

Figure S-19. Drawings of the OLYP DFT-optimized structures of PCBM, iso-PCBM, and faux hawk fullerene (compound **2**). The drawings of PCBM and iso-PCBM were reported in Larson, B. W.; Whitaker, J. B.; Popov, A. A.; Kopidakis, N.; Rumbles, G.; Boltalina, O. V.; Strauss, S. H. "Thermal [6,6] → [6,6] Isomerization and Decomposition of PCBM (Phenyl-C_{61}-butyric Acid Methyl Ester," *Chem. Mater*. **2014**, *26*, 2361–2367 (reference 105 in the main text).

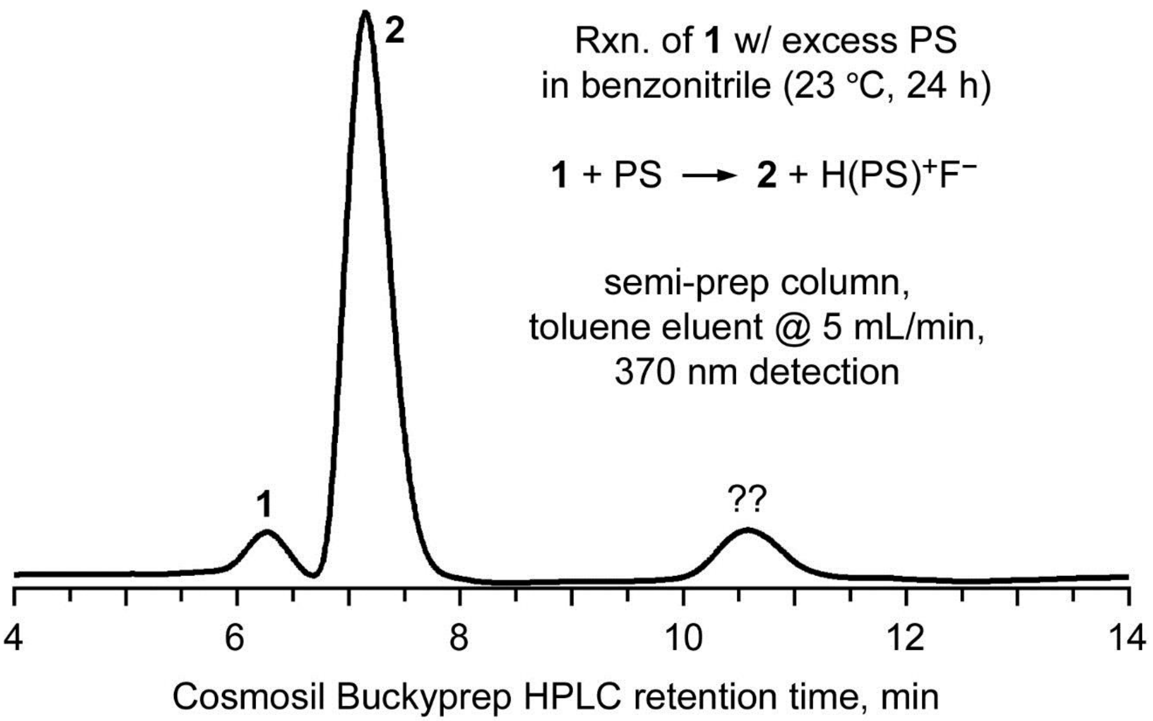

Figure S-20. HPLC trace of the "**1** plus excess Proton Sponge in PhCN" reaction mixture, as described in the Experimental Section in the main text. PS = Proton Sponge; **1** = 1,9-$C_{60}(CF_2C_6F_5)H$; **2** = 1,9-C_{60}(*cyclo*-CF_2(2-C_6F_4)).

References for Supplementary Information

(reference numbers below are different than the numbers in the main text)

1. G. Paternò, A. J. Warren, J. Spencer, G. Evans, V. García Sakai, J. Blumberger and F. Cacialli, *J. Mater. Chem.*, 2013, **1**, 5619.
2. M. Casalegno, S. Zanardi, F. Frigerio, R. Po, C. Carbonera, G. Marra, T. Nicolini, G. Raos and S. V. Meille, *Chem. Commun.*, 2013, **49**, 4525.
3. C. C. Sun, *J. Pharm. Sci.*, 2007, **96**, 1043.
4. R. C. I. MacKenzie, J. M. Frost and J. Nelson, *J. Chem. Phys.*, 2010, **132**, 06904_1.
5. D. L. Cheung and A. Troisi, *J. Phys. Chem. C*, 2010, **114**, 20479.
6. J. H. Choi, T. Honda, S. Seki and S. Fukuzumi, *Chem. Commun.*, 2011, **47**, 11213.
7. H. Oberhofer and J. Blumberger, *Phys. Chem. Chem. Phys.*, 2012, **14**, 13846.
8. A. M. Nardes, A. J. Ferguson, J. B. Whitaker, B. W. Larson, R. E. Larsen, K. Maturová, P. A. Graf, O. V. Boltalina, S. H. Strauss and N. Kopidakis, *Adv. Funct. Mater.*, 2012, **22**, 4115.
9. F. Gajdos, H. Oberhofer, M. Dupuis and J. Blumberger, *J. Phys. Chem. Lett.*, 2013, **4**, 1012.
10. M. T. Rispens, A. Meetsma, R. Rittberger, C. J. Brabec, N. S. Sariciftci and J. C. Hummelen, *Chem. Commun.*, 2003, 2116.
11. N. R. Tummala, S. Mehraeen, Y.-T. Fu, C. Risko and J.-L. Brédas, *Adv. Funct. Mater.*, 2013, **23**, 5800.
12. C. J. Tassone, A. L. Ayzner, R. D. Kennedy, M. Halim, M. So, Y. Rubin, S. H. Tolbert and B. J. Schwartz, *J. Phys. Chem. C*, 2011, **115**, 22564.
13. R. D. Kennedy, M. Halim, S. I. Khan, B. J. Schwartz, S. H. Tolbert and Y. Rubin, *Chem. Eur. J.*, 2012, **18**, 7418.
14. A. F. Wells, *Structural inorganic chemistry*, Oxford University Press, Oxford, 5th ed. edn., 1984.
